# Radiocarbon constraints on the extent and evolution of the South Pacific glacial carbon pool

**DOI:** 10.1038/ncomms11487

**Published:** 2016-05-09

**Authors:** T. A. Ronge, R. Tiedemann, F. Lamy, P. Köhler, B. V. Alloway, R. De Pol-Holz, K. Pahnke, J. Southon, L. Wacker

**Affiliations:** 1 Alfred-Wegener-Institut Helmholtz-Zentrum für Polar- und Meeresforschung, Department for Marine Geology, PO Box 120161, Bremerhaven 27515, Germany; 2 School of Geography, Environment and Earth Sciences, Victoria University of Wellington, PO Box 600, 6012 Wellington, New Zealand; 3 GAIA-Antárctica Universidad de Magellanes, Department of Paleclimatology, Oceanography, Punta Arenas 01855, Chile; 4 Max Planck Research Group—Marine Isotope Geochemistry, Institute for Chemistry and Biology of the Marine Environment, Department of Marine Isotope Geochemistry, Carl von Ossietzky University, PO Box 2503, Oldenburg 26111, Germany; 5 School of Physical Science, Department of Earth Science, University of California, Irvine, California 92697-4675, USA; 6 Laboratory of Ion Beam Physics (HPK), Eidgenössische Technische Hochschule, Schafmattstrasse 20, Zürich 8093, Switzerland

## Abstract

During the last deglaciation, the opposing patterns of atmospheric CO_2_ and radiocarbon activities (Δ^14^C) suggest the release of ^14^C-depleted CO_2_ from old carbon reservoirs. Although evidences point to the deep Pacific as a major reservoir of this ^14^C-depleted carbon, its extent and evolution still need to be constrained. Here we use sediment cores retrieved along a South Pacific transect to reconstruct the spatio-temporal evolution of Δ^14^C over the last 30,000 years. In ∼2,500–3,600 m water depth, we find ^14^C-depleted deep waters with a maximum glacial offset to atmospheric ^14^C (ΔΔ^14^C=−1,000‰). Using a box model, we test the hypothesis that these low values might have been caused by an interaction of aging and hydrothermal CO_2_ influx. We observe a rejuvenation of circumpolar deep waters synchronous and potentially contributing to the initial deglacial rise in atmospheric CO_2_. These findings constrain parts of the glacial carbon pool to the deep South Pacific.

## Abstract (web summary)

On glacial/interglacial timescales CO_2_ might have been sequestered from the atmosphere and stored within the deep ocean. Here, the authors show that an old and CO_2_-rich water mass occupied the glacial South Pacific between ∼2,000 and 4,300 m water depth and was influenced by volcanic CO_2_ from mid-ocean ridges.

## Graphical abstract

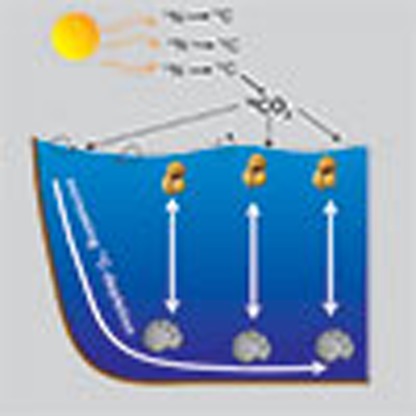

The deep ocean contains the largest carbon reservoir within the global carbon cycle that might interact with the atmosphere on glacial/interglacial timescales. Therefore, the deglacial rise in atmospheric CO_2_ by ∼90 p.p.m.v. (ref. 1) was probably linked to significant modifications in oceanic circulation that resulted in increasing rates of CO_2_ outgassing^2^,^3^,^4^. Thus, in order to sequester large amounts of atmospheric CO_2_, the deep glacial ocean must have been effectively cut off from gas exchange with the atmosphere. Throughout a glacial period, the isolation of deep waters from the surface, and hence the atmosphere, leads to an accumulation of carbon (CO_2_) and nutrients in the deep ocean, which is accompanied by a progressive depletion of radiocarbon (^14^C). Consequently, the older a water mass gets, the more enriched in ^14^C-depleted CO_2_ it becomes.

So far, only isolated occurrences of old glacial water masses have been identified in the North and South Pacific, as well as in the South Atlantic, which suggests that the storage of CO_2_ occurred in the deep glacial ocean^4^,^5^,^6^,^7^,^8^ (Supplementary Fig. 1 and Supplementary Table 1). In particular, the overturning circulation of the Southern Ocean (SO), where nowadays ∼65% of all deep waters make first contact with the atmosphere^9^, controls the ventilation of the oceans interior. However, the surface residence time of upwelled waters before re-subduction is an important factor controlling the efficiency of air–sea gas exchange. Changes in the climate system of the SO, such as the intensification or weakening of stratification or westerly winds have the potential to significantly alter the oceanic uptake or release of CO_2_ (refs 2, 3 and 10) and likewise the radiocarbon budget of deep waters. Therefore, the circum-Antarctic upwelling region is considered the most likely deglacial pathway of stored old carbon from the abyss to the atmosphere. In this oceanic window, carbon-rich deep waters like Pacific Deep Water (PDW) are mixed and upwelled and provide a major source for Antarctic Intermediate Water (AAIW), formed close to the Subantarctic Front (SAF)^11^. Hence, AAIW is able to propagate the circulation- and outgassing signals into the major ocean basins. In this context, numerous intermediate-water records have been analysed to track the timing and pathways of SO deep water upwelling^12^,^13^,^14^,^15^,^16^. The spatial and temporal dimension of the glacial reservoir itself as well as the pathway, magnitude and process of the deglacial CO_2_ release remain elusive, although evidence for carbon storage in the deep glacial southwest Pacific is increasing^5^,^6^.

Here, to better constrain the glacial carbon pool, its vertical extent and evolution, we use Δ^14^C-records from six sediment cores at the New Zealand Margin (NZM; Fig. 1a and Supplementary Fig. 2), covering the major South Pacific water masses AAIW and Upper Circumpolar Deep Water/Lower Circumpolar Deep Water between ∼830 and ∼4,300 m water depth (Fig. 1b). To assess the lateral extent of the glacial carbon pool, we have additionally analysed an open-ocean sediment core from the East Pacific Rise (EPR; PS75/059-2; 3,613 m) located more than 4,000 km east of the NZM (Fig. 1a). Our NZM depth transect is well suited for the analysis of SO water mass ventilation as we can record ^14^C-depleted deep waters on their way to the upwelling region further south, as well as recently subducted intermediate waters moving towards the north (Fig. 1b).

We show that throughout the water column, a wide range of radiocarbon activities (Δ^14^C) indicates a highly stratified South Pacific during the last glacial. This stratification implicates pronounced sequestration of CO_2_ in circumpolar deep waters below a water depth of ∼2,000 m. Building on the hypothesis of increased glacial outgassing of volcanic CO_2_ along mid-ocean ridges (MORs)^17^,^18^,^19^, we use a simple box model to highlight that the most extreme ^14^C-depletion between ∼2,500 and ∼3,600 m water depth might be explained by a combination of aged ^14^C-depleted waters and the additional admixture of ^14^C-dead hydrothermal CO_2_. At the end of the glacial period, our deep water Δ^14^C-values increase throughout the water column in unison to rising atmospheric CO_2_-values^1^. On the basis of these patterns, we conclude that the deep South Pacific was an important contributor to the deglacial rise in atmospheric CO_2_.

## Results

### Radiocarbon

**Figure 1:**
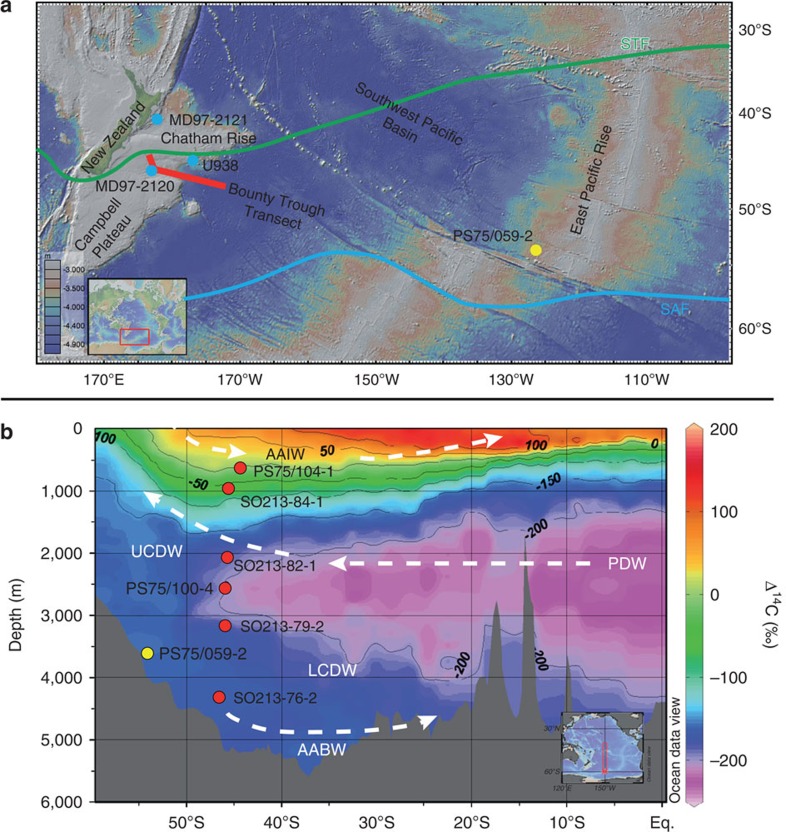
Overview of the NZM showing core locations and water masses. (**a**) Map of the Southwest Pacific. The red bar represents the lateral area covered by our sediment core transect at the NZM. Yellow circle—position of our sediment core from the EPR. Blue circles—previous studies. U938 (ref. 5), MD97–2120 (ref. 15), MD97–2121 (ref. 6). Green line—Subtropical Front. Blue line—Subantarctic Front^66^. Map created using GeoMapApp. (**b**) Water mass section of modern South Pacific Δ^14^C-concentrations. Sediment cores (red and yellow dots) projected into WOCE line P16 (ref. 67). AABW, Antarctic Bottom Water; WOCE, World Ocean Circulation Experiment. Section generated using ODV 4.7.2 (ref. 68).

**Figure 2:**
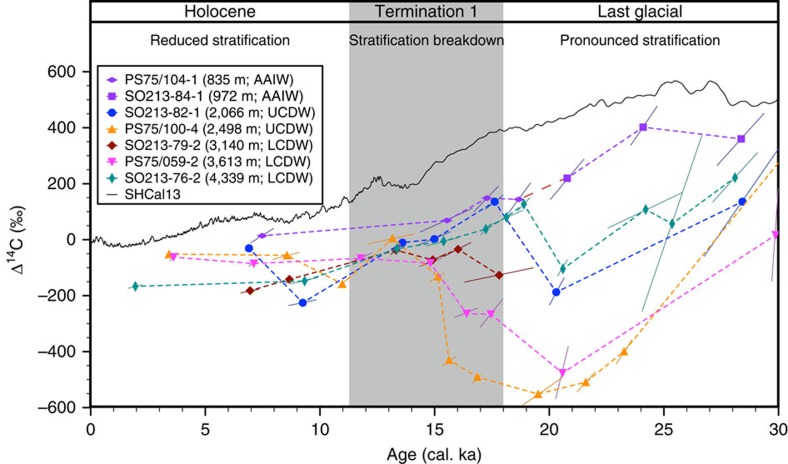
Δ^14^C changes of the major South Pacific water masses. The large glacial range in Δ^14^C indicates a pronounced water mass stratification, followed by a progressive stratification breakdown during Termination 1 (grey shaded area). The broken red line indicates the area, where we spliced PS75/104-1 and SO213-84-1.

To assess both glacial and deglacial ventilation changes, we used paired samples of *Globigerina bulloides* and mixed benthic foraminifers from seven new sediment cores, located south of the present Subtropical Front (STF) (Fig. 1a). The core locations have sedimentation rates between 2.5 cm per kyr and 22 cm per kyr (Supplementary Table 2). Before the Last Glacial Maximum (LGM), ∼29 cal. ka, all deep-water masses (below ∼2,000 m) show Δ^14^C-values ranging from +200‰ to −100‰ (Fig. 2). At the same time, AAIW Δ^14^C is clearly elevated with values of ∼360‰. The most obvious feature of our reconstructed Δ^14^C-values over the LGM is the large glacial range of Δ^14^C between ∼830 and ∼4,300 m (400‰ to −550‰; Fig. 2). About 21 cal. ka, deep-water Δ^14^C at 4,300 and 2,066 m increases, followed by increasing radiocarbon values at 2,500 m at the onset of the last deglaciation. Parallel to increasing deep-water radiocarbon concentrations, AAIW-Δ^14^C slightly decreases. At ∼14.7 cal. ka, the Δ^14^C-records of all water depths converge and continue to evolve parallel to each other throughout the deglaciation and into the Holocene (Fig. 2). In the discussion, we used ΔΔ^14^C-records for our interpretations. These records represent the Δ^14^C offset of our data to the Δ^14^C-value of the past atmosphere^20^. We also calculated the ΔΔ^14^C_adj_ according to Cook and Keigwin^21^ (Supplementary Fig. 3). This method corrects the initial Δ^14^C values to the modern, pre-industrial ^14^C profile (ref. 21). As the trend in our data remains the same, regardless of the method used, we use ΔΔ^14^C for our discussion, in order to improve the comparability to other studies.

### Box modelling

**Figure 3:**
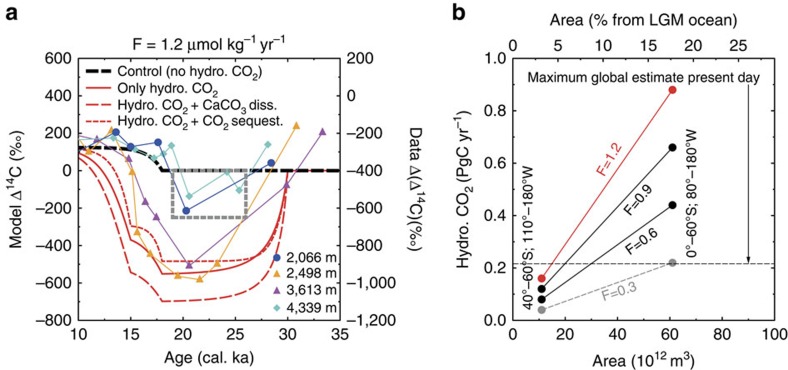
Simulation results of a 1-box model for the effect of hydrothermal CO_2_-outgassing on oceanic Δ^14^C. (**a**) Comparison of model-based Δ^14^C with data-based ΔΔ^14^C. SO213-82-1 (2,066 m; blue line); PS75/100-2 (2,498 m; orange line); PS75/059-2 (3,613 m; pink line); and SO213-76-2 (4,339 m; green line). The impact of our best-guess hydrothermal CO_2_ flux (F) of 1.2 μmol kg^−1^ yr^−1^ between 30 and 15 cal. ka (red solid line) is compared with a control run (black broken line). In the control run, we only decreased the turnover time at 18 cal. ka according to Skinner *et al*.^6^ from 2,700 to 1,500 years. In two sensitivity runs, we estimated the influence of CaCO_3_ dissolution or CO_2_ sequestration (red broken lines). The grey box indicates the ΔΔ^14^C-area covered by previous Southern Ocean studies^4^,^6^,^8^. (**b**) Upscaling of our localized results for different hydrothermal CO_2_ fluxes (F) to regional carbon fluxes as a function of area. Present day maximum global estimate of hydrothermal CO_2_ fluxes (0.22 PgC yr^−1^) (ref. 24) indicated by the black broken line.

We used a 1-box model to investigate the hypothesis that hydrothermal CO_2_-fluxes might have contributed to our reconstructed maximum depletion in Δ^14^C in the glacial deep ocean (Methods). We simulated the single effect of hydrothermal CO_2_ inflow on oceanic Δ^14^C, as well as two sensitivity runs in which hydrothermal CO_2_ inflow is combined with either carbonate compensation, leading to sediment dissolution^22^ or with CO_2_ sequestration, potentially connected with deep-ocean volcanism^23^. Our model simulates for the single effect of different CO_2_ flux rates F (in μmol per kg per year; Supplementary Fig. 4) a drop in Δ^14^C by −240‰ (F=0.3), −380‰ (F=0.6), −480‰ (F=0.9) and −550‰ (F=1.2). Only if the response of the marine carbonate system to this CO_2_ flux (by carbonate compensation and the dissolution of sediments) is considered^22^ (Supplementary Fig. 4), we calculate Δ^14^C amplitudes in agreement with our maximally depleted data (approximately −500‰ to −600‰) for a hydrothermal flux of 0.6 μmol kg per year or larger. However, as hot rocks interact with seawater, MOR volcanism is also discussed as a potential CO_2_ sink^23^. If we implement this process of similar size of the hydrothermal CO_2_ flux (no net oceanic carbon change and therefore no carbonate compensation) we need a hydrothermal CO_2_ flux F of 1.2 μmol kg per year to meet the maximum Δ^14^C depletion of∼−500‰, as observed in our data (Fig. 3a). To convert the CO_2_ fluxes to gross carbon fluxes (PgC per year) we estimated the minimum area covered by our sediment cores as a ∼1,000-m-thick water mass (2,500–3,600 m as covered by PS75/100-4 and PS75/059-2) ranging from 40°S to 60°S and 110°W to 180°W. The fluxes necessary to influence such a water mass would lead to a hydrothermal injection of CO_2_ of 0.08–0.16 PgC per year (modern global flux is estimated to up to ∼0.22 PgC per year)^24^. Upscaling to a larger water mass, spanning most of the South Pacific (1,000 m; 0°–60°S; 80°–180°W) would imply that the hydrothermal CO_2_ flux might be as large as 0.44–0.88 PgC per year (Fig. 3b).

## Discussion

**Figure 4:**
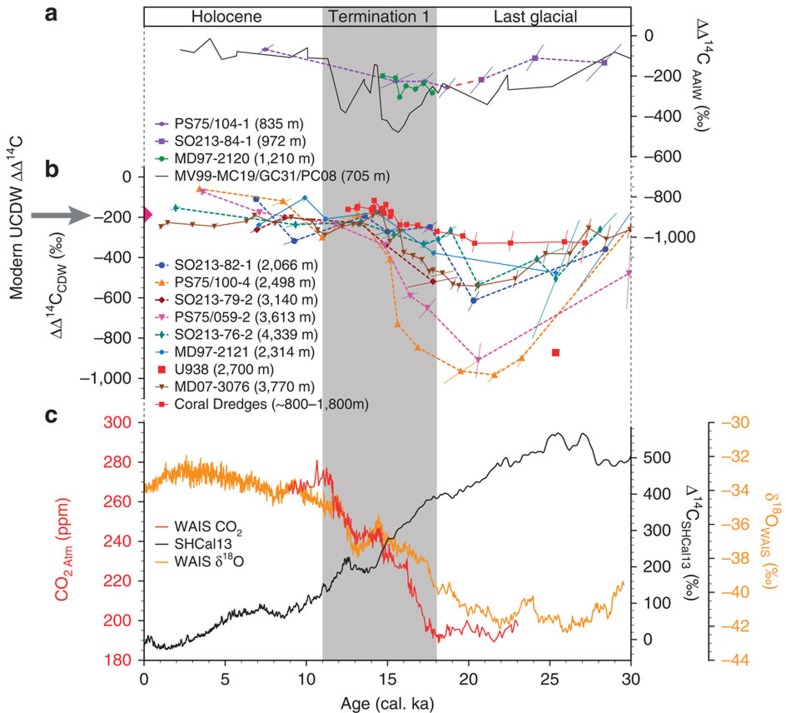
Comparison of oceanic and atmospheric proxy records. ΔΔ^14^C-values (ocean–atmosphere) of (**a**) the intermediate Pacific region; PS75/104-1 and SO213-84-1 (this study); MD97–2120 (ref. 15) (green line: Bounty Trough); MV99-MC19/GC31/PC08 (ref. 12) (black line: Northeast Pacific); (**b**) CDW ΔΔ^14^C; PS75 and SO213 records (this study); MD97–2121 (ref. 6) (light blue line: north of Chatham Rise); U938^5^ (red square: Bounty Trough); Coral dredges^8^ (red line: Drake Passage); MD07-3076 (ref. 4 (brown line: South Atlantic); and Modern SW-Pacific UCDW ΔΔ^14^C (ref. 67) (pink triangle). (**c**) Atmospheric CO_2_ concentrations^1^ (red line); WAIS δ^18^O record^69^ (orange line) and atmospheric Δ^14^C-values^20^ (black line). UCDW, Upper Circumpolar Deep Water.

**Figure 5:**
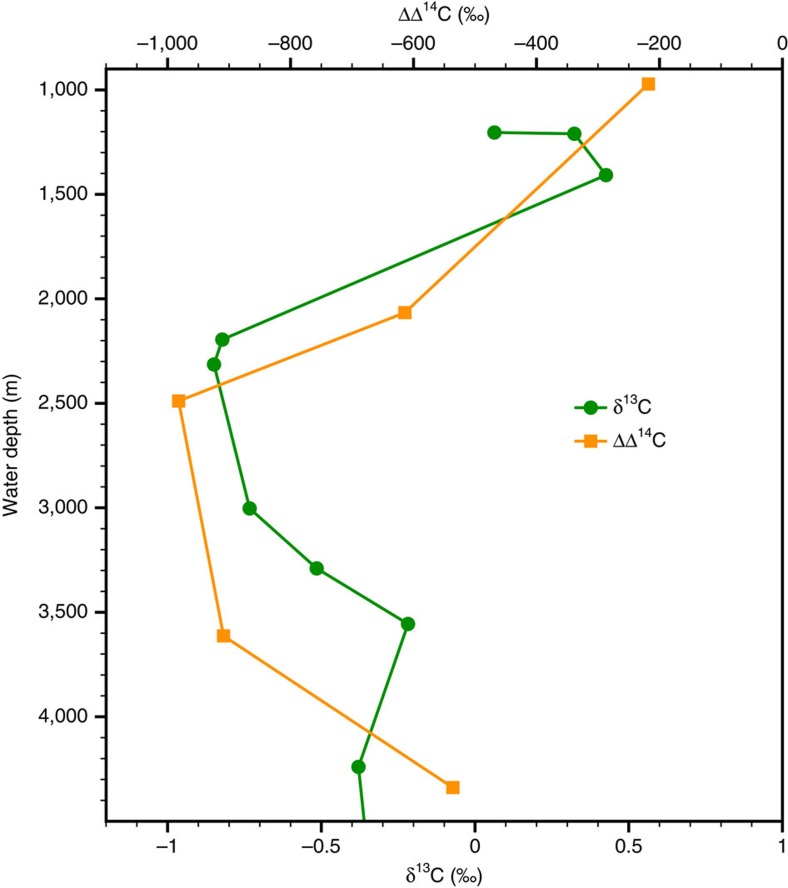
Vertical distribution of carbon isotopes off New Zealand throughout the LGM. Orange line—ΔΔ^14^C (this study). Green line δ^13^C_*Uvigerina*_^27^.

Throughout the water column, the observed glacial Δ^14^C-range in our cores exceeds the modern and Holocene values by a factor of ∼5 and indicates strong age differences and therefore enhanced stratification of the intermediate and deep glacial South Pacific. Along its pathway in the global thermohaline circulation PDW is fed into circumpolar waters and constitutes today's oldest water mass. The ^14^C-depleted PDW presently extends to 2,000–2,500 m north of the Chatham Rise close to New Zealand^25^ (Fig. 1b). From our transect, we are able to show that during the LGM, significantly ^14^C-depleted and aged water masses occupied depths between ∼2,000 and ∼4,300 m in the Southern Westerly (SW) Pacific (Fig. 2). Our data locate the core of the ^14^C-depleted water mass at the NZM in a of ∼2,500 m (PS75/100-4; modern depth of Upper Circumpolar Deep Water; Fig. 4), yielding a maximum deep water to atmosphere offset in radiocarbon activities (ΔΔ^14^C) of approximately −1,000‰. Analysing ΔΔ^14^C corrects for any impacts of changes in ^14^C production^26^ as well as for variable ocean–atmosphere exchange rates. A corresponding apparent ventilation age, based on benthic minus reservoir-corrected planktic ^14^C ages would equate to ∼8,000 years (Supplementary Fig. 3d). A similar glacial ΔΔ^14^C depletion of about −870‰ was reported from sediment core U938, which was recovered at the NZM in a water depth of 2,700 m (ref. 5) (Figs 1a and 4). We hypothesize that these extremely low ΔΔ^14^C-values might be the result of the admixture of ^14^C-dead hydrothermal CO_2_ into a water mass with an initial high ventilation age, which was estimated to at least 2,700 years^6^. The upper and lower boundary of this old water mass are marked by higher ΔΔ^14^C-values of −550‰ to −600‰ indicating a highly stratified water column (Fig. 4b). Similar ΔΔ^14^C-values were reported at the NZM north of Chatham Rise at 2,314 m (ref. 6). This confines the most radiocarbon-depleted waters to a depth below ∼2,300 m. The observed trend of ^14^C between 830 and 4,300 m parallels the highest glacial nutrient concentrations off New Zealand, between 2,000 and 3,000 m (ref. 27) (Fig. 5), likewise indicative for the presence of aged, nutrient rich (low δ^13^C) and radiocarbon-depleted waters. Yet, the δ^13^C reconstructions might yield a certain bias, as endobenthic (*Uvigerina*) instead epibenthic (*Cibicidoides*) foraminifera were used^27^.

We traced the ^14^C-depleted glacial carbon reservoir off New Zealand to the central South Pacific (EPR) 4,000 km east of the NZM. At this location, ΔΔ^14^C-values are as negative as −900‰ at 3,600 m (PS75/059-2; Figs 1a and 4). Hence, we are confident that this water mass was not only restricted to the NZM but seems to have occupied large parts of the South Pacific. Further off, in the Drake Passage and the South Atlantic, glacial water masses have been identified in CDWs with ΔΔ^14^C-values as low as −330‰ (ref. 8) and −540‰ (ref. 6), respectively (Fig. 4). In their timing and amplitudes, these records are similar to our Pacific radiocarbon signature characterizing the upper and lower boundary of the old carbon pool (Fig. 4b). Our intermediate-water record (SO213-84-1; modern depth of AAIW) shows the highest glacial ΔΔ^14^C-values of our transect (approximately −90‰). We suggest that the ^14^C-depleted deep waters represent the very old return flow from the North Pacific (PDW), similar to the modern circulation pattern (Fig. 1b). The distribution of radiocarbon in our reconstruction might indicate a floating carbon pool instead of a stagnant bottom layer. Several records from the North Pacific might corroborate this assumption. In the Gulf of Alaska^28^ (MD02-2489) and off Kamchatka^29^ (MD01-2416), as well, the glacial mid-depth water mass (Kamchatka) shows a considerable higher benthic to planktic ^14^C offset than the deeper water mass off Alaska. Additional data from the northwest Pacific suggest the lowest glacial ^14^C values in a water depth of ∼2,300 m with better ventilated waters above and below^21^. In the Atlantic Ocean as well, Ferrari *et al*.^30^ and Burke *et al*.^31^ observed a mid-depth (floating) radiocarbon anomaly. A floating carbon pool might furthermore explain why no sign of old carbon was found in the deep equatorial Pacific below 4,000 m (ref. 32). Therefore, this record might have ‘missed' the old, mid-depth carbon pool above.

**Figure 6:**
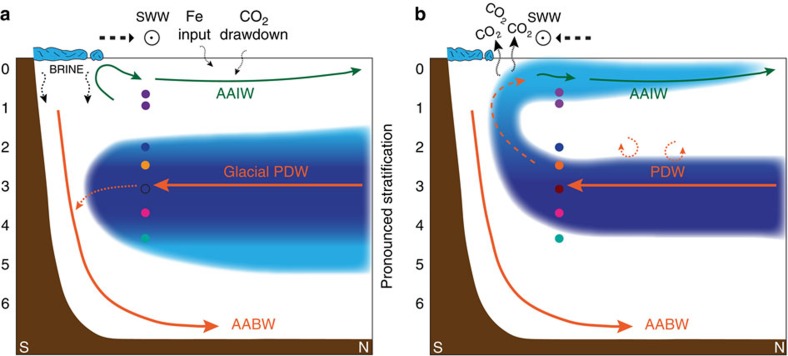
Schematic representation of South Pacific overturning circulation. (**a**) Glacial pattern: northernmost extent of sea ice and SWW. Increased AABW-salinity by brine rejection favours stratification. Increased dust input promotes primary production and drawdown of CO_2_. (**b**) Deglacial pattern: upwelling induced by southward shift of Antarctic sea ice and SWW. The erosion of the deep-water carbon pool releases ^14^C-depleted CO_2_ towards the atmosphere. Following air–sea gas exchange, the outgassing signal is incorporated into newly formed AAIW (light blue shading). Blue shading: poorly ventilated old and CO_2_-rich waters; Darkest shading 2,500–3,600 m: water level influenced by hydrothermal CO_2_. Green arrows: intermediate water; orange arrows: deep-water; light-blue areas: sea ice; SWW: Southern Westerly Winds; coloured circles: sediment cores (colour coding according to Fig. 2); black circle: SO213-79-2—no glacial data; and circular arrows: diffusional and diapycnal mixing.

Any explanation for the pronounced glacial radiocarbon-depletion of the deep SO has to involve a limited ocean–atmosphere exchange due to strengthened ocean stratification under glacial boundary conditions^3^ (Fig. 6a). Northward-expanded Antarctic sea ice and SW Winds^33^ contributed to reduced air–sea gas exchange and upwelling of deep-waters^30^. Surface freshening by melting sea-ice in the source regions of intermediate waters^34^ and enhanced formation of highly saline Antarctic Bottom Water^35^ may have set a density structure that led to reduced mixing and the encasement of old PDW (Fig. 6a). In addition, the shoaling of North Atlantic Deep Water^30^ might have reduced the contribution of freshly ventilated waters into South Pacific CDW below ∼2,000 m. These interacting key processes may have significantly contributed to the low radiocarbon values and are consistent with an enhanced glacial storage of carbon in the deep ocean.

**Figure 7:**
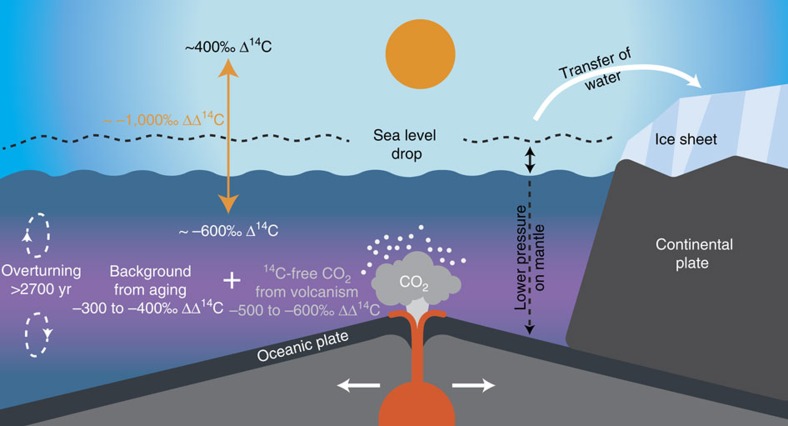
Processes linking the glacial release of hydrothermal CO_2_ and water mass Δ^14^C. The drop in global sea level triggers increased volcanic activity at MORs. The plume of ^14^C-dead hydrothermal CO_2_ is mixed into an aged water mass, in which the combined effects of surface reservoir age and deep-ocean turnover time, of ∼2,700 years, led already to a background ocean-to-atmosphere offset in ΔΔ^14^C of −300‰ to −400‰. This admixture of hydrothermal CO_2_ further lowers the water masses ΔΔ^14^C by another −500‰ to −600‰, yielding a total ΔΔ^14^C of about −1,000‰ (purple layer). Modified after Hand^70^. Reprinted with permission from AAAS.

**Figure 8:**
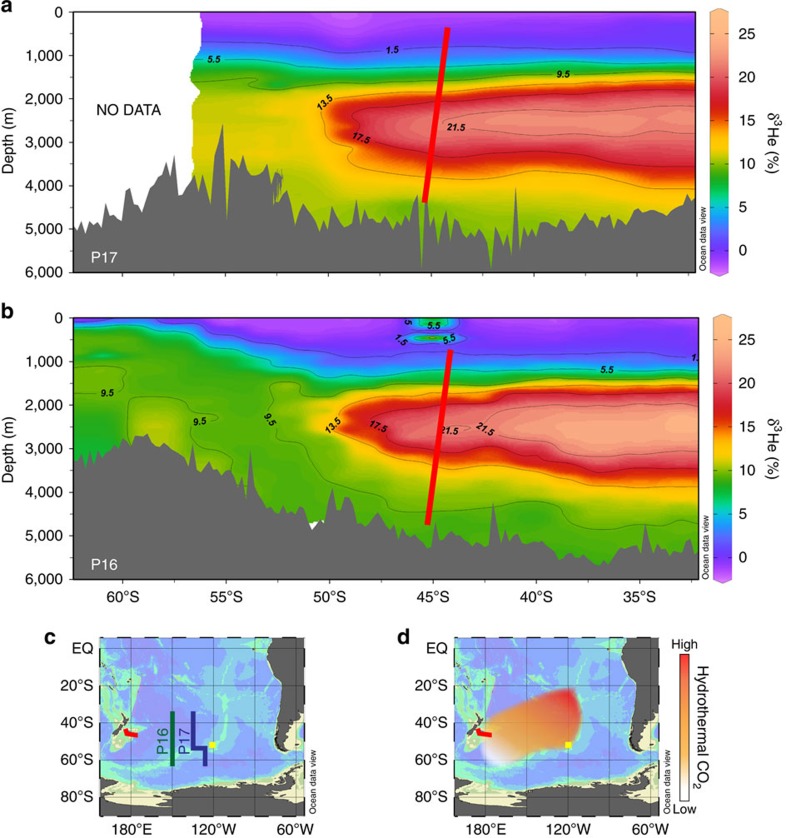
Dispersal of hydrothermal ^3^He in the Southwest Pacific. The modern hydrothermal ^3^He plume, emanating at the southern EPR in a water depth of ∼2,500 m (refs 38 and 39), can be traced throughout the southern Pacific towards New Zealand^41^. ^3^He distribution along (**a**) WOCE line P17 (ref. 67) (∼135° W) and (**b**) WOCE line P16 (ref. 67) (150° W). (**c**) Locations of ^3^He sections P16 and P17. (**d**) Hypothesized dispersal of the glacial hydrothermal plume emanating from the EPR. Core locations and depths indicated by red bars and yellow squares. Panels generated using ODV 4.7.2 (ref. 68). WOCE, World Ocean Circulation Experiment.

Old water masses of 5,000–8,000 years are expected to be strongly oxygen depleted^36^. According to Sarnthein *et al*.^7^, water masses with Δ^14^C values lower than −350‰ would be completely anoxic. However, pronounced anoxia have not been documented in the deep South Pacific between 2,500 and 3,600 m. Therefore, an admixture of ^14^C-dead carbon via submarine tectonic activity along MOR^18^ into a an old water mass in the deep South Pacific might have contributed to the extremely low radiocarbon values of the water mass at ∼2,500–3,600 m. During the LGM, sea floor eruption rates along tectonically active plate boundaries may have intensified due to the lower glacial sea level^17^,^18^,^19^. This process might have released significant amounts of ^14^C-dead CO_2_ into the water column. Using a simple 1-box model (Methods), we tested our hypothesis and calculated if the injection of hydrothermal CO_2_ into the deep Pacific has the potential to amplify the ΔΔ^14^C minimum throughout the LGM (Fig. 3). To overcome the influence of the variable atmospheric ^14^C-levels^26^,^37^, we compared our simulated Δ^14^C to our reconstructed ΔΔ^14^C values (deep ocean-to-atmosphere offset). The probably time-delayed response of submarine volcanism to changes in sea level complicate our flux calculations^19^. A crucial prerequisite for our hypothesis of the admixture of hydrothermal CO_2_ is the presence of an already aged water mass with high nutrient concentrations and low Δ^14^C levels (Fig. 7). According to the record of MD97–2121 (ref. 6) (2,314 m), the glacial ventilation age off New Zealand is at least 2,700 years. However, radiocarbon values might have been even lower as the MD97–2121 record lacks any data points between ∼25 kyr and ∼18 cal. ka (Fig. 4b). Our ΔΔ^14^C record of SO213-82-1 (2,066 m; Fig. 4b) is −600‰ at ∼20kyr, ∼100‰ lower than the minimum observed in MD97–2121 ∼25 cal. ka, potentially indicating even higher turnover times. When we combine the radioactive decay, caused by an estimated water mass age of 2,700 years for the time of 35–18 cal. ka, with submarine ^14^C-free volcanic CO_2_ influx, our model calculates a decrease in Δ^14^C for the corresponding water mass by additional −500‰ to −600‰ (Fig. 3a). This hydrothermal CO_2_ outgassing (potentially accompanied by carbonate compensation and/or CO_2_ sequestration) would lead to a maximum atmosphere-to-deep ocean offset of −800‰ to −1,000‰ ΔΔ^14^C (Supplementary Fig. 4e–g), comparable to the maximum depletion observed in PS75/059-2 and PS75/100-4. Today, in a water depth between ∼2,500 and ∼3,500 m, pronounced volcanic outgassing occurs along the southern EPR^38^,^39^. The resulting hydrothermal plume spreads towards the west and can be traced by the ^3^He-signal in the broader western Pacific (Fig. 8)^40^ and off northern New Zealand, right in the water depth under debate of ∼2,500 m (ref. 41). Therefore, we argue that increased glacial outgassing of ^14^C-dead volcanic CO_2_ into a stratified ocean has the potential to significantly lower the Δ^14^C-content of an old (at least 2,700 years) water mass. The prominent Chatham Rise (Fig. 1a) might have acted as a physical barrier, blocking MD97–2121 (ref. 6) (∼2,300 m) from the volcanic plume. As MD97–2121 lacks data for most of the last glacial (∼18–25 cal. ka), we cannot fully exclude that this core might have been affected by volcanic CO_2_ to some extent. Nevertheless, the ^14^C-data of MD97–2121 are already significantly higher at ∼18 cal. ka compared to the values of PS75/100-4. Therefore, we argue that the influence (if any) of hydrothermal activity must have been lower to the north of the Chatham Rise and/or at 2,300 m water depth. Despite the in detail unknown processes accompanying such a hydrothermal carbon flux, its admixture might add additional carbon to the ocean–atmosphere–biosphere system (0.08–0.16 PgC per year; Fig. 3b). However, the net carbon injection depends in detail on the strength of the additional processes carbonate compensation and CO_2_ sequestration and might also be zero. Once the glacial processes, favouring stratification, are reversed, any net injected carbon might eventually be released to the atmosphere along with the carbon already stored within the deep ocean (Fig. 5b). A further quantification of the net carbon injection and its contribution to atmospheric CO_2_ is not yet possible, since future investigations with process-based models are necessary. Furthermore, as the distribution of MOR is inhomogeneous in the world ocean, it is difficult to compare our local results to global CO_2_ flux estimates^24^. In Fig. 3b, we illustrate that the water mass affected by hydrothermal CO_2_ might span an area of between 3 and 17% of the global glacial ocean. While the results for the minimum area, covered by our sediment cores, are below the maximum estimate of present day global estimate of hydrothermal outgassing, the results for an area representative for most of the South Pacific are a factor of 2–4 times higher (Fig. 3b). This suggests that if the admixture of hydrothermal CO_2_ is the process that can explain the minimum ΔΔ^14^C values recorded in the mid-depth South Pacific (PS75/100-4; PS75/059-2; U938 (ref. 5)) the global CO_2_-fluxes from MOR throughout the LGM might have been much larger than today. However, if the initial water mass was older than the 2,700 years assumed in our model, the resulting fluxes might also have been smaller than stated here. Although mantle-CO_2_ is depleted in both, Δ^14^C and also in δ^13^C (−5±3‰ (refs 42 and 43)), its influence might be stronger on radiocarbon. As ^14^C is by far less common in the ocean than ^13^C, it can be diluted (lowered) more easily than its non-radiogenic counterpart.

We assume that the previously outlined glacial/interglacial changes in the SO climate system (position of sea ice and westerlies; changes in water mass densities; changes in upwelling and circulation) are the major factors influencing the spatio-temporal evolution of the oceanic carbon pool. However, the extreme minima in ΔΔ^14^C between 2,500 and 3,600 m water depth in the South Pacific are most plausibly explained by the hypothesized admixture of hydrothermal CO_2_ into an old and already ^14^C-depleted water mass. Admittedly, this process would complicate the use of ^14^C as a ventilation proxy for the water masses affected by hydrothermal CO_2_. However, as the presence of an already existing ^14^C-depleted glacial water mass is a crucial prerequisite for our model, our theory does not interfere with the concept of stratification, decreased ventilation and the presence of a glacial oceanic carbon pool.

In the Pacific, other processes affect the radiocarbon inventory of water masses as well. Stott and Timmermann^44^ already suggested the release of ^14^C-depleted carbon from gas clathrates. As the stability of such clathrates is located in shallow waters ∼400 m (ref. 44), this process is not applicable for our mid-depth anomaly below ∼2,500 m. Therefore, we reject the possibility of any large influence of ^14^C-depleted CO_2_ and CH_4_ clathrates.

At the end of the LGM and during the transition into the Holocene (∼20–11.5 cal. ka), converging ΔΔ^14^C-values argue for a progressive destratification (Fig. 4). During this interval, the deep-water-to-atmosphere offset in radiocarbon between ∼2,000 and ∼4,300 m decreases significantly (Fig. 4b). Although the resolution of our deep-water sediment cores is rather low, their ΔΔ^14^C-values increase within error synchronous to the rise in atmospheric CO_2_ rise (Fig. 4).

During Termination 1, when the most radiocarbon-depleted deep waters rejuvenate, no pronounced depletion in AAIW ΔΔ^14^C is recorded (Fig. 4a). However, the intermediate-water ΔΔ^14^C-values remain low from ∼18–15 cal. ka. The decrease in the deep water-to-atmosphere ΔΔ^14^C-offset and the abrupt drop in δ^13^C of atmospheric CO_2_ (ref. 45) suggests increased air–sea gas exchange and the oceanic release of upwelled old CO_2_ (Fig. 5b). The intermediate-waters from the NZM (this study) and the Chile Margin^14^ significantly deviate from the (sub)tropical East Pacific, which shows two prominent drops in ΔΔ^14^C at the intermediate-water level during Termination 1 (refs 12, 13) (Fig. 4a). Therefore, it seems unlikely that southern sourced AAIW represents the source for the deglacial radiocarbon signals in the (sub)tropical East Pacific.

As we mentioned before, because of the close proximity, similar reservoir ages for all sediment cores are a requirement for our interpretations. However, the Holocene reservoir ages of two of our cores differ by ∼1,000 years (Supplementary Fig. 5). Although this offset does not change the overall story, it prevents us from discussing the data of this time interval in more detail.

Our reconstructions provide new insights into the evolution and dynamic of the marine carbon inventory, its aging and the process of CO_2_ release to the atmosphere. Growing evidence throughout large parts of the glacial South Pacific (this study; Skinner *et al*.^6^), the North Pacific^21^,^28^,^29^, the Drake Passage^8^ and the South Atlantic^4^ suggest the existence of a floating body of very old, ^14^C-depleted water between 2,000 and 4,300 m water depth, particularly in the tectonically active Pacific Ocean, where the glacial admixture of volcanic CO_2_ might have influenced the ^14^C-signature of this carbon pool from ∼2,500 to ∼3,600 m water depth. The effect of such volcanic CO_2_ flux on the global carbon cycle as a whole and on atmospheric CO_2_ in particular needs to be assessed in future studies, using more sophisticated models. During Termination 1, the atmosphere to deep-water ΔΔ^14^C of the carbon reservoir was reduced from about −1,000‰ to about −200‰ (Fig. 4b), indicating the erosion of the glacial carbon pool. This erosion is in accordance with the concept of a deglacial breakdown of SO stratification^3^,^45^,^46^ and intensified deglacial wind-driven SO upwelling^2^. These processes ultimately culminated in the release of ^14^C-depleted CO_2_ from the deep ocean reservoir to the atmosphere (Fig. 5b), although in detail, the interaction of processes affecting the biological and physical pumps—and thus atmospheric CO_2_—was probably more complex^47^.

## Methods

### Sediment core details and sample treatment

The water mass transect that forms the backbone of this study consists of seven sediment cores retrieved during the ANTXXVI/2 and SO213/2 cruises from the Bounty Trough off New Zealand (NZM) and from the EPR (Supplementary Figs 1 and 2). Collectively, these sediment cores record all water masses between 835 and 4,339 m, thus the modern water depths of the AAIW down to the Lower Circumpolar Deep Water with an imprint of Antarctic Bottom Water^27^. Positions, water depth, modern water masses and average sedimentation rates are reported in Supplementary Table 2.

An advantage of the NZM core transect is that it was not affected by potential glacial/interglacial shifts of the STF or the SAF^48^,^49^. The STF is bathymetrically fixed by the Chatham Rise (Supplementary Fig. 2), while the SAF is topographically steered by the submerged Campbell–Bounty Plateau^48^,^49^. As all sediment cores were located south of the STF and because of their close proximity to each other, we assume that changes in surface reservoir ages would affect all locations in a similar way. An exception is core PS75/059-2, which was retrieved ∼4,200 km east of the Bounty Trough, at the western flank of the EPR (Supplementary Fig. 1) ∼2°N of the SAF.

All sediment cores were split to form working and archival halves. The working half was sampled at 2 cm intervals. Depending on their water content, all samples were freeze dried for 2–3 days. Subsequently, the samples were wet sieved, using a 63 μm mesh sieve and dried at 50 °C for 2 days. As a last step, all samples were subdivided into the size fractions >400 μm, 315–400 μm, 250–315 μm, 125–250 μm and <125 μm. Benthic and planktic foraminifera were picked from 250 to 315 μm and 315 to 400 μm size fractions, taking great care to group similar sized individuals into samples for isotope analyses.

For the analysis of AAIW ventilation, we spliced sediment cores PS75/104-1 (835 m) and SO213-84-1 (972 m). We were forced, to combine both records as PS75/104-1 did not yield a sufficient amount of benthic foraminiferal fauna below a core depth of ∼100 cm (LGM and older), while SO213-84-1 was significantly disturbed above ∼50 cm core depth (Termination 1 and younger).

### Radiocarbon measurements

For the reconstruction of radiocarbon activities, corresponding pairs of planktic (monospecific *G. bulloides*) and benthic (mix of *Cibicidoides wuellerstorfi* and *Uvigerina peregrina*) foraminifera were picked. To minimize the effect of contamination on our analyses, we paid special attention not to pick any broken, discoloured or filled tests. Radiocarbon measurements were performed at the National Ocean Science Accelerator Mass Spectrometer (NOSAMS) facility in Woods Hole, USA, the W. M. Keck Carbon Cycle AMS Laboratory at the University of California in Irvine, USA and at the Laboratory for Ion Beam Physics at the Eidgenössische Technische Hochschule in Zurich, Switzerland.

We calculated the difference in benthic and reservoir-corrected planktic (B-P) ^14^C-ages to reconstruct the apparent ventilation ages of different water masses and compared our reconstructions with the method proposed by Cook and Keigwin^21^ (Supplementary Fig. 4).

The equation of Adkins and Boyle^50^ was used to determine the initial (paleo) radiocarbon activity (Δ^14^C) of the benthic samples.

The difference of deep-water Δ^14^C to the contemporaneous past atmospheric Δ^14^C (called ΔΔ^14^C) is the offset of our data from the IntCal13 reference curve^20^.

Despite a potential bias by uncertainties in our age models and the IntCal13 (ref. 20) curve, we show in Supplementary Fig. 6 that the trend in ΔΔ^14^C remains the same, regardless of the method used.

The radiocarbon dates for all cores are stored in the PANGAEA-database. Ventilation age errors were calculated from combined errors in ^14^C ages and calibrated calendar ages. The error in Δ^14^C was calculated from ^14^C and calibrated age errors. All ^14^C-data can be found in Supplementary Table 3 and on the PANGAEA-database.

### Age control

For all sediment cores, an initial radiocarbon chronology was obtained from planktic ^14^C-datings. Planktic radiocarbon ages were calibrated to calendar ages, using the calibration software Calib 7.0 (refs 51 and 52) with the embedded SHCal13 calibration curve^20^. To account for surface reservoir effects, we corrected all ^14^C-ages according to reservoir age estimates by Skinner *et al*.^6^. However, to allow the use of ^14^C as an unbiased proxy for deep-water ventilation, we fine-tuned our records to the nearby reference core MD97–2120 (ref. 53) (Supplementary Figs 7–9). We chose MD97–2120 (1,210 m water depth) as a reference core because of its position close to our sediments cores in the Bounty Trough. The original stratigraphy of MD97–2120 (refs 53 and 54) is based on nine ^14^C measurements in the time interval between 0–35 kyr. Following the method applied by Rose *et al*.^15^, we correlated the planktic δ^18^O and Mg/Ca-derived SST records of MD97–2120 (ref. 54) with the EDC ice core δD record^55^,^56^ (Supplementary Fig. 7). Correlating MD97–2120 via surface temperatures to Antarctic ice cores enables us to obtain a ^14^C-independent stratigraphy that we can use as the baseline for the X-ray fluorescence (XRF) core-to-core correlation.

At the Alfred Wegener Institute in Bremerhaven, Germany, all sediment cores were analysed for their specific element abundances using an Avaatech XRF core-scanner with a preset step width of 1 cm. The resulting element content records (Sr, Ca, Fe and Sr/Fe) were then correlated to respective age-scaled XRF records of the sediment core MD97–2120 (refs 53 and 54) from the southern Chatham Rise (Supplementary Fig. 8) using the computer program AnalySeries 2.0.4.2. In particular, the Sr-counts were useful for the core-to-core correlation, as Sr represents CaCO_3_, but is barley affected by differences in grain sizes or the sediments water-content^57^. Furthermore, we were able to utilize a distinctive vitric-rich tephra layer as a radiocarbon-independent stratigraphic marker within two sediment cores (SO213-76-2 and SO213-79-2). The tephra layer in both cores was geochemically characterized and identified as the widespread Kawakawa/Oruanui tephra (KOT; for analytical details see the section on tephra analyses). The KOT is a widespread silicic tephra erupted from Taupo Volcanic Centre in the central North Island of New Zealand with an age of ∼25.36 cal. ka (ref. 58) and is the most important isochronous tephra marker erupted in the SW-Pacific region during the past 30,000 years^59^. This tephra was likewise found in reference core MD97–2120 (ref. 54). We adjusted the KOT age used by Pahnke *et al*.^54^ to the revised age by Vandergoes *et al*.^58^.

The age model of the EPR-core PS75/059-2 is based on the original approach of Lamy *et al*.^60^. However, as this age model lacks detailed tie-points between 0 and 30 cal. ka, we fine-tuned PS75/059-2 to the age-scaled record PS75/100-4, using their Sr XRF element counts. Despite a certain lack in the LGM variability of most XRF-records, we consider our age models as relatively robust. In particular the comparison of our records SO213-82-1 and SO213-76-2 to records from similar water depths in the South Pacific^6^ (MD97–2121), the Drake Passage^8^ (coral dredges) and the South Atlantic^4^ (MD07–3076), reveals a similar timing as well as comparable ΔΔ^14^C-values (Fig. 4) for all records.

The errors for the correlated age models were estimated from the offset to the ^14^C-derived age model plus ^14^C-errors.

Our XRF-based correlation method creates new surface reservoir ages for our sediment records. These differ from the surface reservoir age record of MD97–2121 (ref. 6), but are still in good agreement with these reconstructions (Supplementary Fig. 5). However, owing the XRF-correlation method, our reservoir ages show a slightly higher scatter than the records of Sikes *et al*.^5^ and Skinner *et al*.^6^

### Tephra analyses

In cores SO213-76-2 (507–508 cm) and SO213-79-2 (150–151 cm) a distinctive vitric-rich tephra layer was identified. Morphological expression, grain size and thickness of this tephra were indistinguishable and hence, most likely to represent the same eruptive event. Glass shard major element chemistry of this tephra layer was then determined by electron microprobe analysis and the results were compared with selected onshore and offshore KOT correlatives (Supplementary Fig. 10). The glass shard geochemistries were consequently indistinguishable which (a) affirms correlation to KOT and (b) augments the overall chronology of this study. All major element determinations were made on a JEOL Superprobe (JXA-8230) housed at Victoria University of Wellington, using the ZAF correction method. Analyses were performed using an accelerating voltage of 15 kV under a static electron beam operating at 8 nA. The electron beam was defocused between 10 and 20 μm. All elements calculated on a water-free basis, with H_2_O by difference from 100%. Total Fe expressed as FeO_t_. Mean and ±1 s.d. (Supplementary Table 4; in parentheses), based on *n* analyses. All samples normalized against glass standards VG-568 and ATHO-G.

### Box modelling

Here we make some first-order estimates investigating the hypothesis of an impact of a potential hydrothermal CO_2_ flux on the marine carbonate system and on Δ^14^C in the South Pacific. We simulate carbon cycle changes in a 1-box model water mass, which might represent the mid-depth South Pacific waters ∼2,500–3,500 m (Supplementary Fig. 4a). We start our carbon cycle simulations from an LGM state that was previously simulated with the BICYCLE model^46^. We thus perturb a water mass with dissolved inorganic carbon (DIC) concentration of ∼2,600 μmol kg^−1^, with a total alkalinity of 2,667 μmol kg^−1^. For LGM conditions (temperature ∼0 °C; salinity=35.8 PSU), these factors would lead to a pH of 7.7 and a carbonate ion concentration of ∼70 μmol kg^−1^.

We base our calculations on changes in concentrations for an undefined volume of the water mass and increase the amplitude of hydrothermal CO_2_ until the ^14^C-anomaly, seen in our data, is reproduced by our most simplistic model.

Similar to our data from 2,066 m (SO213-82-1), Skinner *et al*.^6^ provide an independent glacial ventilation age of ∼2,700 years, which decreases to ∼1,500 years at 18 cal. ka. To obtain a stable Δ^14^C of 0‰ in our water mass ∼30 cal. ka (Fig. 3a, black broken line), the incoming carbon flux from X_i_ from oceanic mixing processes has a Δ^14^C signature of +328‰. Corresponding to the decrease in ventilation age from 2,700 to 1,500 years, this flux increases at 18 cal. ka from 1 to 1.7 μmol per kg per year (Supplementary Fig. 4c). The outgoing carbon flux X_o_, leaving our simulated 1-box water mass via oceanic transport processes, is determined by the turnover/ventilation time *τ*. X_o_ might change over time in size due to hydrothermal CO_2_ injection, causing a rise in DIC within the water parcel. For the time window of minimum sea level^61^ (Supplementary Fig. 4b) between 30 and 15 cal. ka, we assume a hydrothermal CO_2_ flux (F) of 0, 0.3, 0.6, 0.9 or 1.2 μmol per kg per year (Supplementary Fig. 4d–g). Please note that this approach assumes an instantaneous feedback of the hydrothermal CO_2_ outgassing rate to the removed load from the drop in global sea level and neglects any potential time delay in the solid earth response. Time delay of this process were briefly discussed previously^18^ but might also be more complex^19^.

On millennial timescales, the injection of hydrothermal CO_2_ might lead to a partly dissolution of CaCO_3_ in oceanic sediments^22^,^62^. Via this process of carbonate compensation, carbonate ions are brought into solution and added to the DIC pool. In this dissolution flux (D), the ratio of alkalinity and DIC is 2:1. If enough CaCO_3_ is available for dissolution, the additional dissolution flux from carbonate compensation is on the order of 88% of the initial CO_2_ injection^63^. In case of insufficient amounts of available CaCO_3_, carbonate ion concentration and pH would fall. So far, it is estimated that the amount of dissolvable sediments is restricted to ∼1,600 PgC (ref. 64), although simulation studies show that the actual dissolution for large CO_2_-injections is smaller than this^22^. While the actual ^14^C signature of dissolved CaCO_3_ is not readily known, we assume the dissolution of ^14^C-free sediment as the upper limit. This assumption is supported by the existence of very old surface sediments in the pelagic Southeast Pacific off Chile^65^. For simplicity, we here calculate an instantaneous additional impact of carbonate compensation. However, since this process is in reality time delayed with an e-folding time of a few thousand years^22^, the most likely solution lies between both scenarios, with and without carbonate compensation.

In addition, we estimate the impact on Δ^14^C, if CO_2_, of similar size (S) as the hydrothermal injection flux (F), is directly sequestered via magmatic processes^23^, leading to stable DIC concentrations.

Please note that the three processes considered here (hydrothermal CO_2_ flux F; carbonate dissolution D; CO_2_ sequestration S) are roughly estimated due to their potential impact on oceanic Δ^14^C. In detail, these processes might be time-delayed or offset from each other.

### Code availability

The computer code for the 1-box model used to calculate the simulation results shown in Fig. 3 and Supplementary Fig. 4 is available upon request from one of the co-authors (PK; peter.koehler@awi.de).

## Additional information

**How to cite this article:** Ronge, T. A. *et al*. Radiocarbon constraints on the extent and evolution of the South Pacific glacial carbon pool. *Nat. Commun.* 7:11487 doi: 10.1038/ncomms11487 (2016).

## Supplementary Material

Supplementary Information Supplementary Figures 1-10, Supplementary Tables 1-4 and Supplementary References.
