# Corrigendum: Radiocarbon constraints on the extent and evolution of the South Pacific glacial carbon pool

**DOI:** 10.1038/ncomms11885

**Published:** 2016-06-03

**Authors:** T. A. Ronge, R. Tiedemann, F. Lamy, P. Köhler, B. V. Alloway, R. De Pol-Holz, K. Pahnke, J. Southon, L. Wacker

Nature Communications 7: Article number: 11487 10.1038/ncomms11487 (2016); Published: 05092016; Updated: 06032016

The affiliation details for R. De Pol-Holz are incorrect in this Article. The correct affiliation details for this author are given below:

GAIA-Antartica, Universidad de Magallanes, Punta Arenas 01855, Chile.

