# Supplementary material for: Radiocarbon constraints on the extent and evolution of the South Pacific glacial carbon pool
Source: Nat Commun. 2016 May 9;7:11487. doi: 10.1038/ncomms11487 (PMC4865812; doi:10.1038/ncomms11487)
Supplement: Supplementary Information — Supplementary Figures 1-10, Supplementary Tables 1-4 and Supplementary References. [file ncomms11487-s1.pdf]

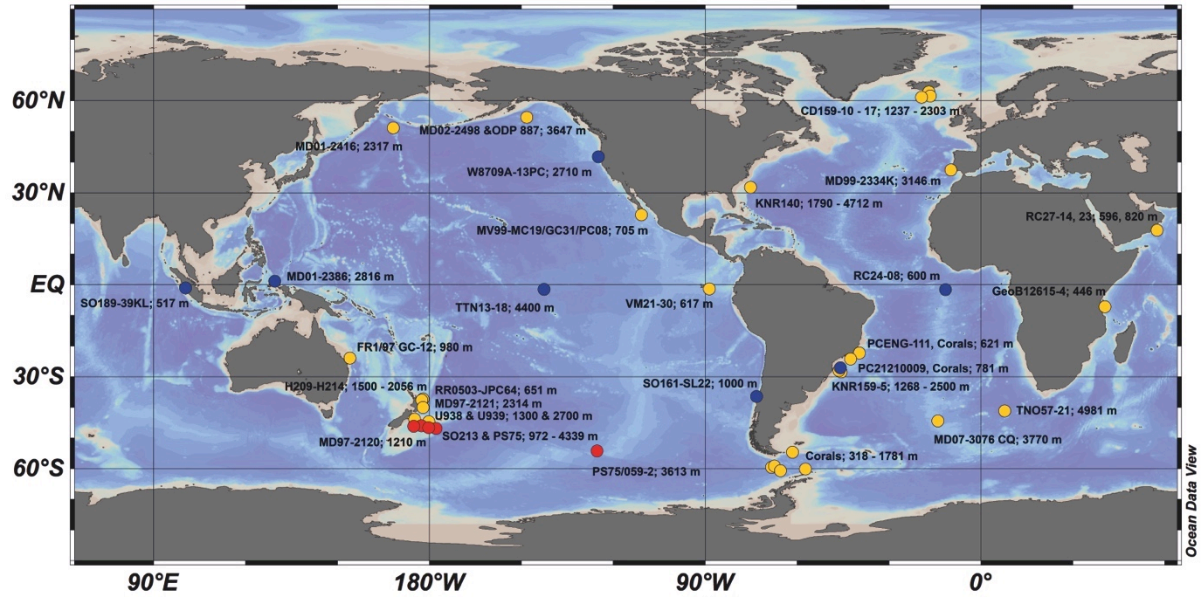

Supplementary Figure 1: Overview map of worldwide sediment cores used to constrain the glacial carbon pool or its pathways. Red dots – this study; orange dots – evidence for old glacial/deglacial water; blue dots – no significantly aged water. See Supplementary Table 1 for core references. Map generated using ODV 4.7.2[1].

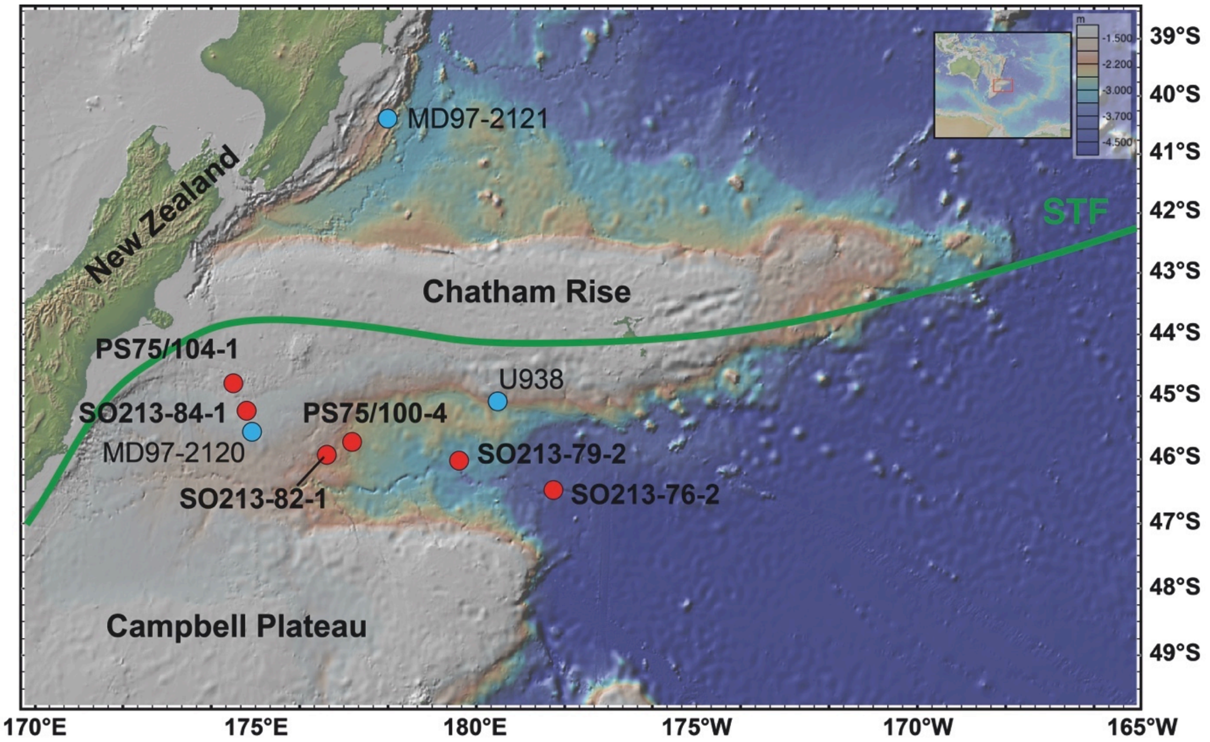

Supplementary Figure 2: **Bathymetric map of the New Zealand Margin.** Core locations used in this study (red dots), previous studies (blue dots; U938[2]; MD97-2120[3]; MD97-2121[4]); Subtropical Front[5] (STF - green line) and major topographic features. Map generated using GeoMapApp (www.geomapapp.org).

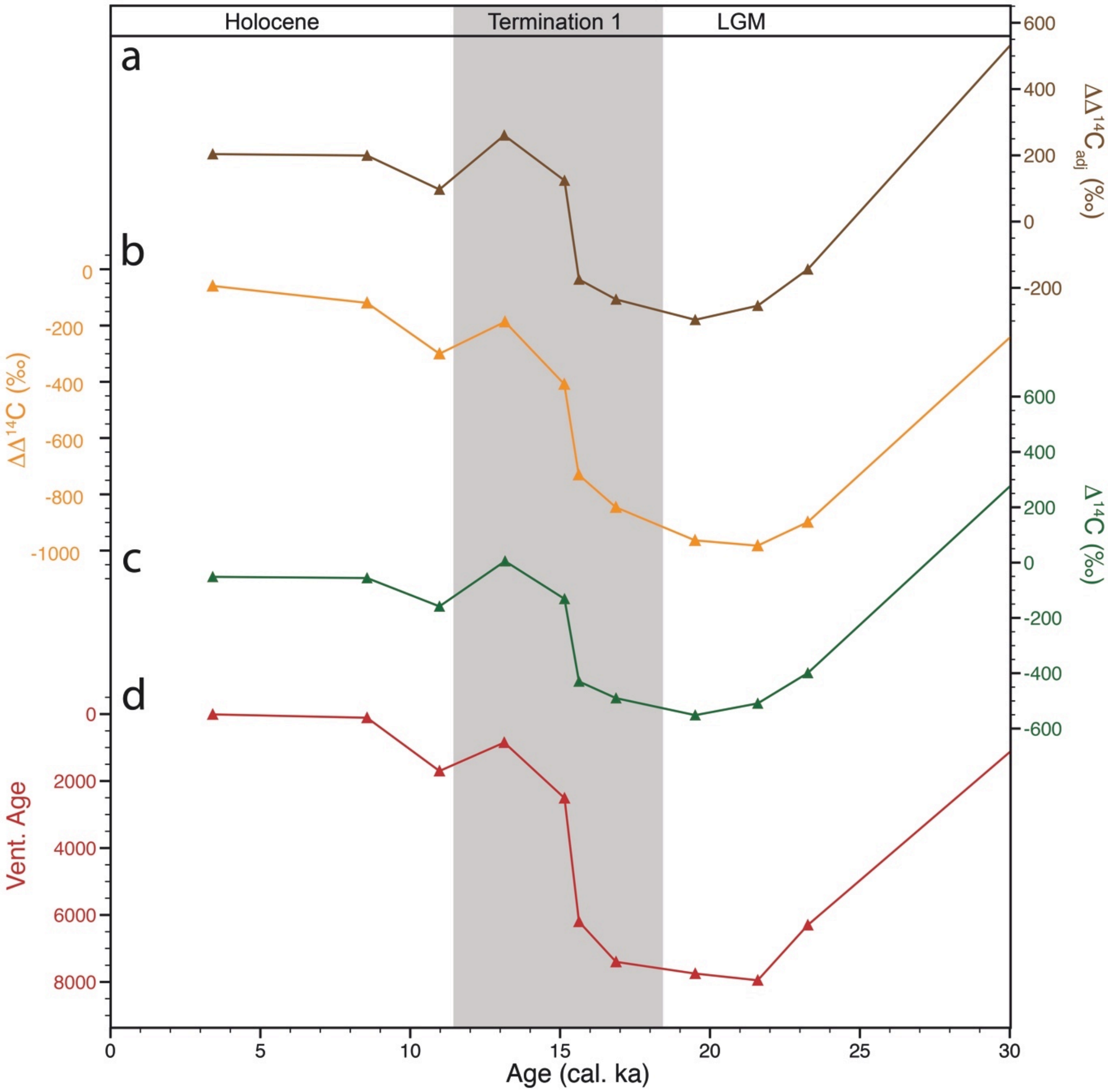

Supplementary Figure 3: Comparison of calculated ^{14}C-parametes of sediment core PS75/100-4 (2498 m). The comparison shows that no matter of the applied method, the general trend in our data remains the same, while absolute values differ. **(a)** $\Delta\Delta^{14}C_{adj}$ according to Cook and Keigwin [6]. **(b)** $\Delta\Delta^{14}C$ as used in the main article. **(c)** Calculated initial $\Delta^{14}C$ according to Adkins and Boyle[7]. **(d)** Apparent deep-water ventilation ages, when hydrothermal input of ^{14}C-dead CO_2 is omitted. Calculated from the ^{14}C age difference of benthic to reservoir corrected planktic foraminifera.

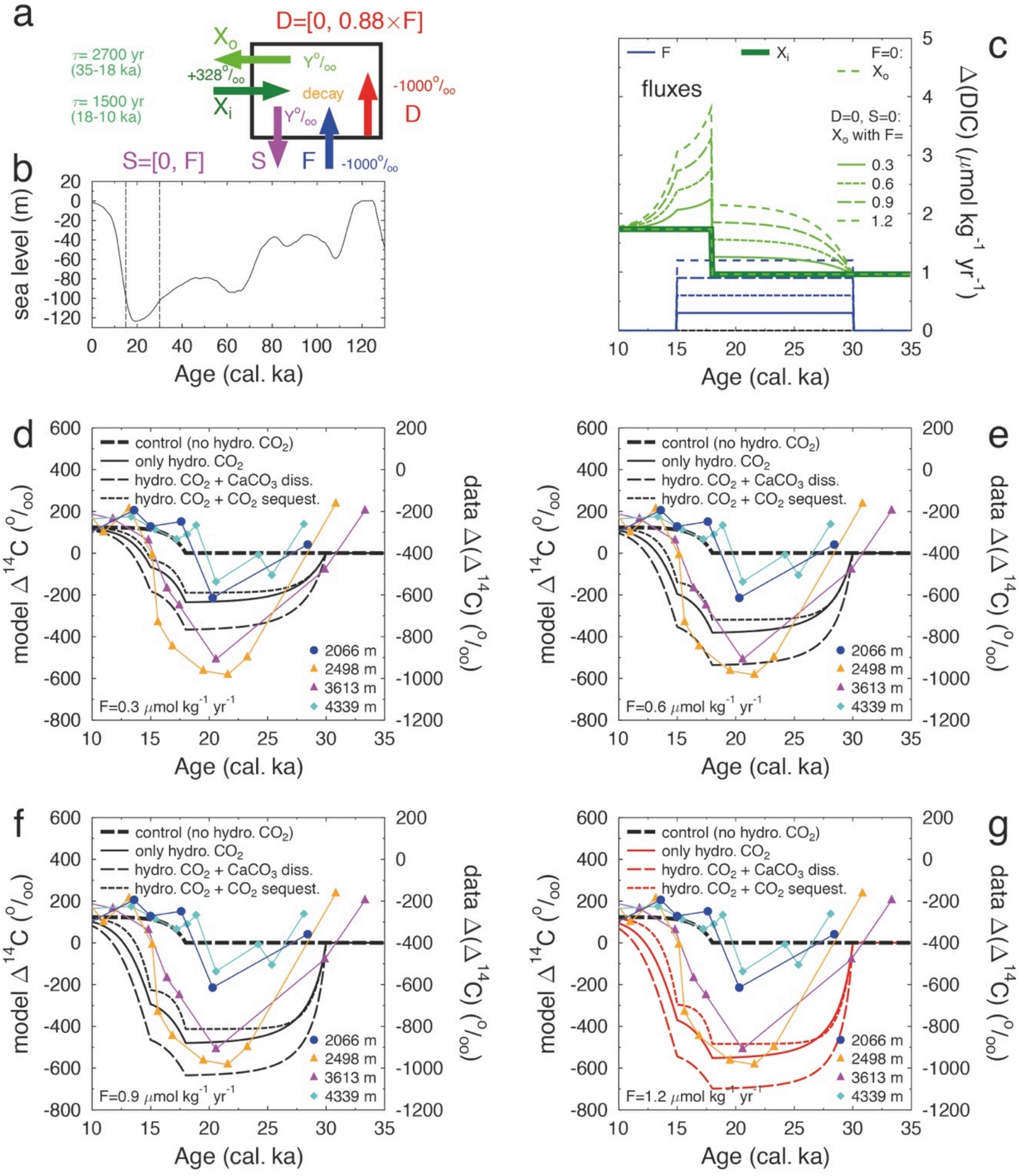

Supplementary Figure 4: Simulation results of a 1-box model for the effect of hydrothermal CO_2 outgassing on $\Delta^{14}C$. **(a)** Schematic of 1-box-model including the ^{14}C signature of all fluxes with ^{14}C of the outgoing exchange flux consisting of the ^{14}C signature Y of the box, thus being variable over time. Hydrothermal CO_2 outgassing (F), carbonate dissolution (D), CO_2 sequestration by hot rock-water interaction (S), exchange with surrounding water masses (X) defined by the turnover times τ[Ref.4]. Radioactive decay is calculated with a half-life time of 5730 years. **(b)** Sea level change during the last glacial cycle[8]. The two vertical lines mark the low stand between 15 and 30 cal. ka. **(c)** Forcing of our 1-box model. Changes in carbon exchange with surrounding waters (incoming flux X_i, outgoing flux X_o), and CO_2 flux due to hydrothermal outgassing (F). F=0.3, 0.6, 0.9 and 1.2 μmol kg^{-1} yr^{-1} **(d-g)** Comparison of model based $\Delta^{14}C$ with data based $\Delta\Delta^{14}C$. The use of data $\Delta\Delta^{14}C$ eliminates the impact of the variable ^{14}C production rate on our interpretations. The coloured symbols indicate sediment records from different water depths (blue – SO213-82-1; orange – PS75/100-4; pink –

PS75/059-2; green – SO213-76-2). The flux rates (in µmol kg^{-1} yr^{-1}) differ in each subfigure (d: F=0.3; e: F=0.6; f: F=0.9; g: F=1.2). In each subfigure, the control run, where only an increase in ventilation rate at 18 cal. ka is considered (corresponding to a decrease in turnover time from 2700 years to 1500 years[4]), is indicated by the black bold broken line. The influence of $CaCO_3$ dissolution or CO_2 sequestration is estimated with two sensitivity runs (thin broken lines). The scenario in subfigure (g) is highlighted by red lines, as this subfigure is also shown in the main text (Fig. 3a).

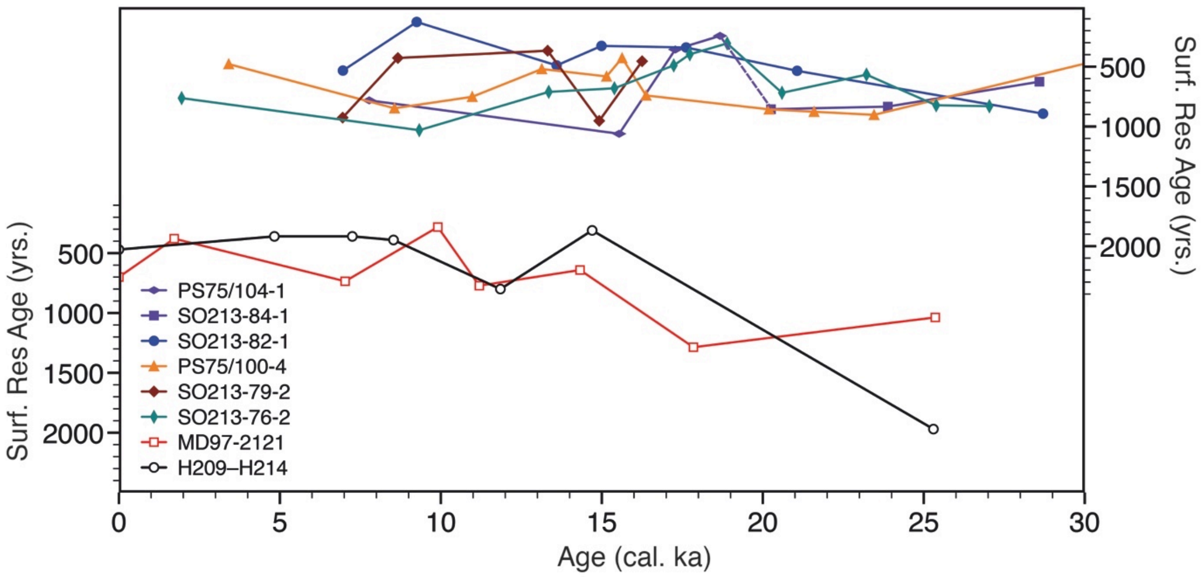

Supplementary Figure 5: Surface reservoir ages of NZM sediment cores. Top panel – this study; bottom panel – previous studies. MD97-2121[4]; H209 to H214[2]. As we spliced PS75/104-1 and SO213-84-1, we connected both records via a broken purple line.

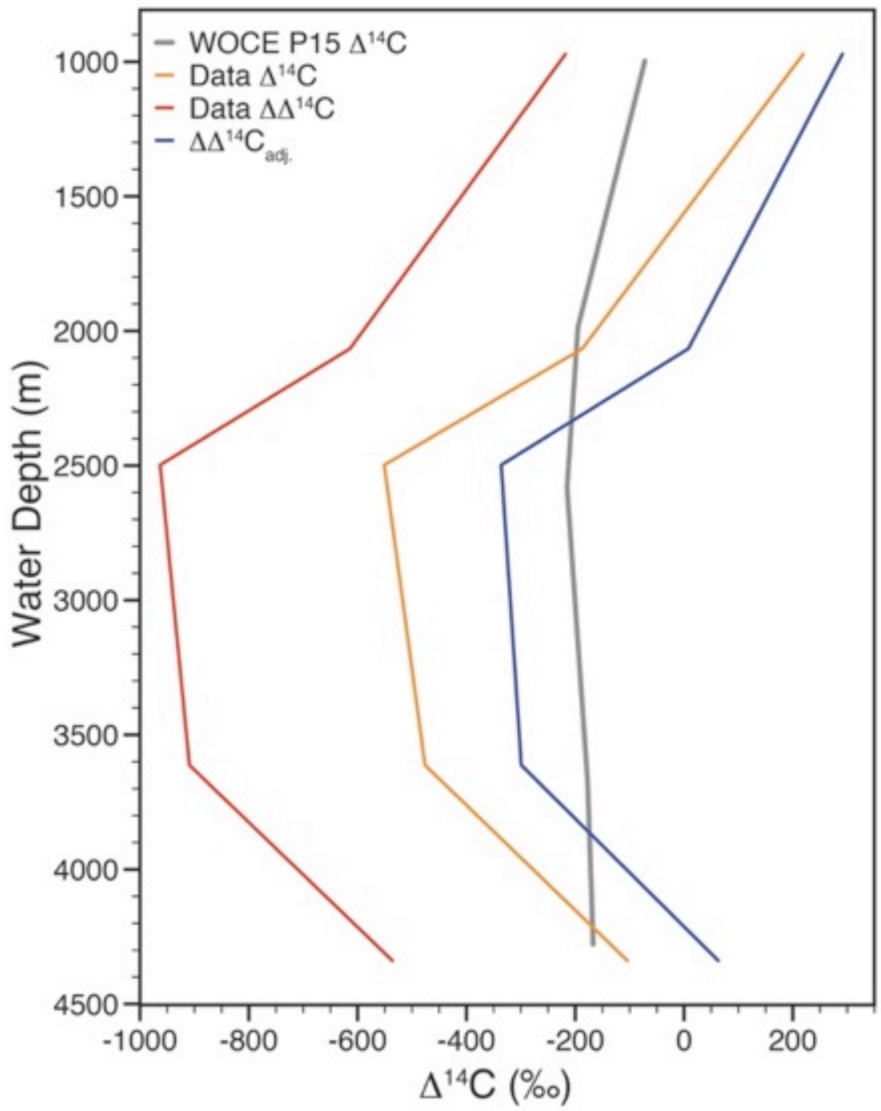

Supplementary Figure 6: Comparison of modern South Pacific $\Delta^{14}C$ (grey[9]) and glacial $\Delta^{14}C$ (orange), $\Delta\Delta^{14}C$ (red) to $\Delta\Delta^{14}C$adj (blue) calculated according to Cook and Keigwin[6].

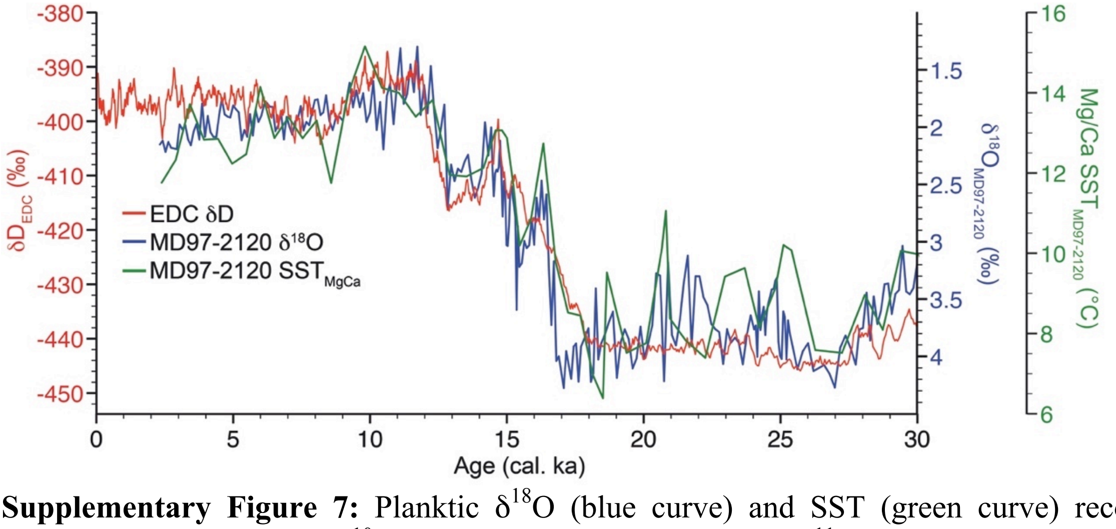

Supplementary Figure 7: Planktic $\delta^{18}O$ (blue curve) and SST (green curve) records of sediment core MD97-2120[10] correlated to the EDC δD record[11] on the AICC12 time scale[12] (red curve).

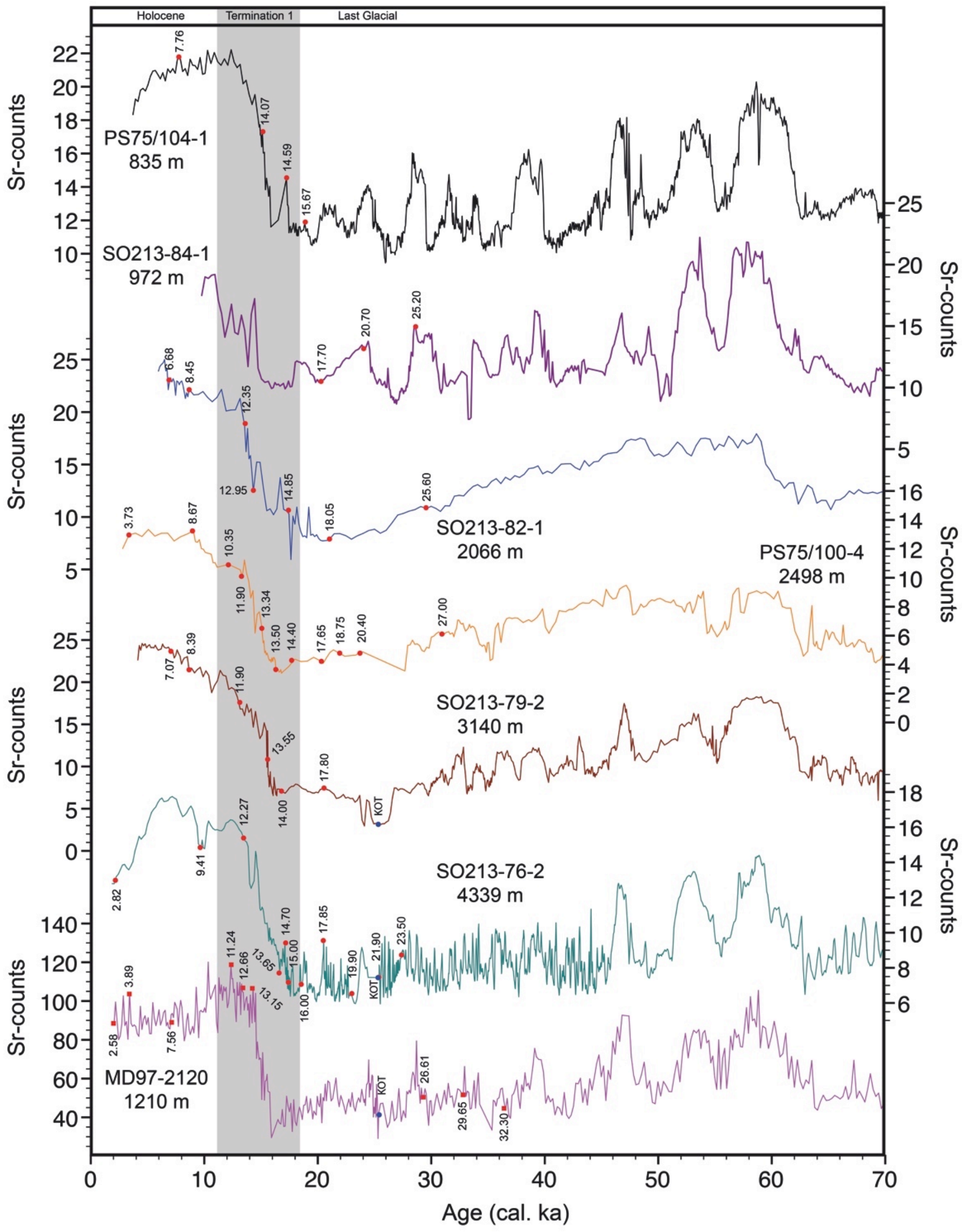

Supplementary Figure 8: XRF correlation of all sediment cores to the reference core MD97-2120 for the past 70 kyr. Displayed here are solely Sr-count curves (scale ∗1000). Likewise, Ca-, Sr/Fe- and Fe-counts, stable isotope records, and tephra analyses were used to constrain the initial ^{14}C-based correlation. Red circles indicate ^{14}C-sampling positions of this study; red squares are sampling positions by Pahnke and Zahn[13]. Black numbers indicate raw ^{14}C ages. YD – Younger Dryas; ACR – Antarctic Cold Reversal; HS1 – Heinrich Stadial 1; LGM – Last Glacial Maximum; Blue circles & KOT – Kawakawa/Oruanui Tephra. Please note that no corresponding benthic ^{14}C age exists for the last sample of SO213-79-2.

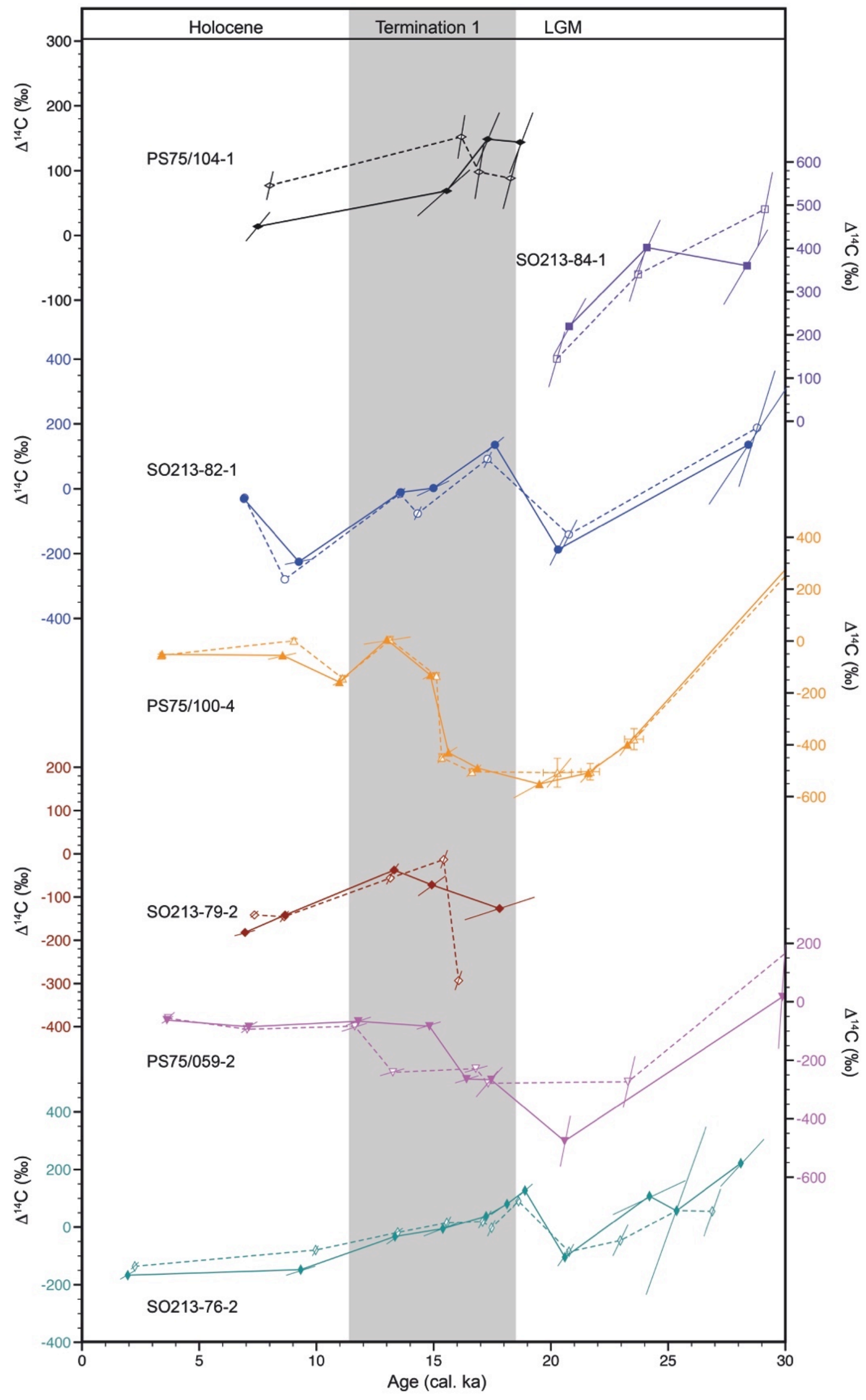

Supplementary Figure 9: Comparison of $\Delta^{14}C$ records generated using different age models. The solid lines are based on the XRF-correlation-method, while the broken lines are based on calibrated planktic ^{14}C ages.

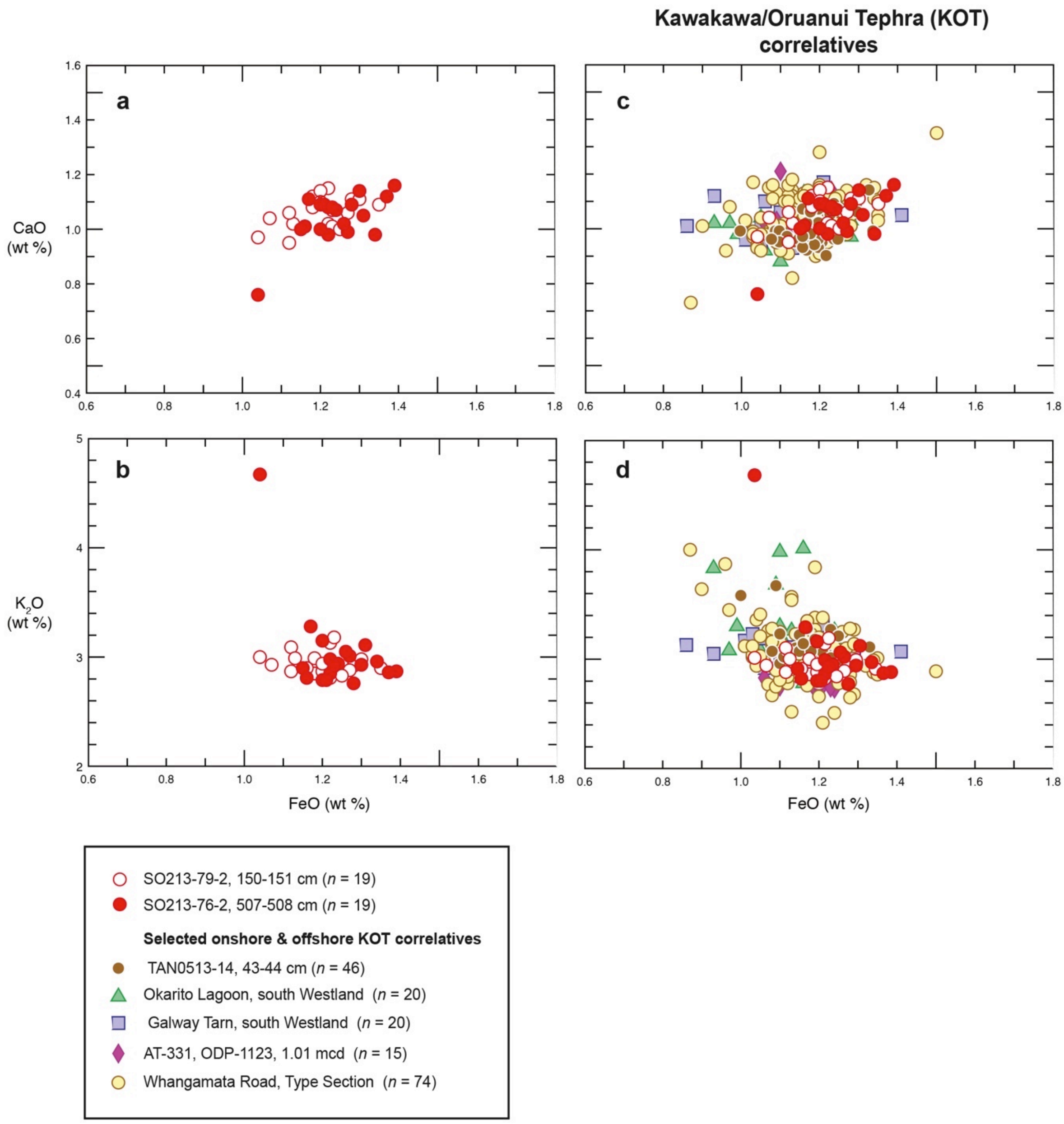

Supplementary Figure 10: CaO **(a)** and K_2O **(b)** to FeO weight percent values from SO213-79-2 (red circles) and SO213-76-2 (red filled circles) in comparison to elemental weight percent values of reference samples of the Kawakawa/Oruanui Tephra (KOT) (**c** and **d**). References for KOT correlatives as indicated for Supplementary Table 3.

Supplementary Table 1: Core details for sediment cores shown in Supplementary Figure 1

Archive	Water Depth (m)	Latitude	Longitude	Reference
TTN13-18	4400	S2°	W140°	Broecker and Clark, Ref. 18
TNO57-21	4981	S41° 1' 0.001"	E7° 7' 59.999"	Barker et al., Ref. 19
MV99-MC19/GC31/PC08	705	N23° 4' 59.999"	W111° 6' 0"	Basack et al., Ref. 20
FR1/97 GC-12	990	S23° 34' 36.998"	E153° 47' 34.001"	Bostock et al., Ref. 21
MD01-2386	2816	N1° 7' 48"	E129° 47' 34.8"	Broecker et al., Ref. 22
RC27-14	596	N18° 3' 0"	E57° 6' 0"	Bryan et al., Ref. 23
RC27-23	820	N18° 0' 3.6"	E57° 6' 0"	Bryan et al., Ref. 23
LMG06-06	318	S54° 29' 6"	W62° 12' 50.4"	Burke and Robinson, Ref. 24
NBP0805	819	S60° 10' 55.2"	W57° 50' 2.4"	Burke and Robinson, Ref. 24
NBP0806	1134	S60° 32' 49.2"	W65° 56' 56.4"	Burke and Robinson, Ref. 24
NBP0807	869	S59° 43' 58.8"	W68° 44' 34.8"	Burke and Robinson, Ref. 24
NBP0808	695	S59° 43' 22.8"	W68° 52' 51.6"	Burke and Robinson, Ref. 24
NBP0809	1750	S59° 42' 25.2"	W69° 0' 28.8"	Burke and Robinson, Ref. 24
NBP0810	978	S59° 44' 34.8"	W68° 53' 52.8"	Burke and Robinson, Ref. 24
NBP0811	1323	S59° 43' 55.2"	W68° 55' 58.8"	Burke and Robinson, Ref. 24
NBP0812	816	S54° 44' 2.4"	W62° 12' 57.6"	Burke and Robinson, Ref. 24
RC24-08	600	S1° 19' 59.999"	W11° 54' 0"	Cleroux et al., Ref. 25
SO161-SL22	1000	S36° 13' 0.001"	W73° 40' 0.001"	De Pol-Holz et al., Ref. 26
SO189-39KL	517	S0° 46' 59.999"	E99° 55' 0.001"	De Pol-Holz et al., Ref. 27
ODP 887	3647	N54° 37' 0.001"	W148° 45' 0"	Galbraith et al., Ref. 28
Coral	1125	S59° 7' 0.001"	W68° 7' 0.001"	Goldstein et al., Ref 29
KNR 140	2900	N32° 7' 0.001"	W75° 42' 0"	Keigwin et al., Ref. 30 & Robinson et al., Ref. 31
W8709A-13PC	2710	N42° 1' 0.001"	W125° 7' 59.999"	Lund et al., Ref. 32
Corals	621	S22° 4' 0.001"	W40° 1' 0.001"	Mangini et al., Ref. 33
Corals	781	S24° 3' 0"	W43° 1' 59.999"	Mangini et al., Ref. 33
MV99-MC19/GC31/PC08	705	N23° 4' 59.999"	W111° 6' 0"	Marchitto et al., Ref. 34
MD02-2489	3640	N54° 23' 11.4''	W148° 50' 499''	Rae et al., Ref. 35
Coral	1125	S59° 4' 0.001"	W68° 4' 59.999"	Robinson et al., Ref. 36
GeoB12615-4	446	S7° 8' 17.999"	E39° 50' 26.999"	Romahn et al., Ref. 37
MD97-2120	1210	S43° 32' 3.599"	E174° 55' 50.999"	Rose et al., Ref. 3
RR0503 JPC 64	651	S37° 25' 20.399"	E177° 0' 24.599"	Rose et al., Ref. 3
MD01-2416	2317	N51° 16' 0''	E167° 44' 0''	Sarnthein et al., Ref. 38
H209	1675	S37° 9' 29.999"	E177° 44' 24"	Sikes et al., Ref. 2
H211	1500	S37° 18' 11.999"	E177° 21' 29.999"	Sikes et al., Ref. 2
H213	2056	S37° 2' 30.001"	E177° 10' 30"	Sikes et al., Ref. 2
H214	2045	S36° 55' 30"	E177° 26' 30.001"	Sikes et al., Ref. 2
U938	2700	S45° 4' 30"	E179° 29' 53.999"	Sikes et al., Ref. 2
U939	1300	S44° 29' 42"	E179° 30' 0"	Sikes et al., Ref. 2
MD99-2334K	3146	N37° 48' 0"	W10° 10' 0.001"	Skinner et al., Ref. 39
MD07- 3076 CQ	3770	S44° 4' 27.599"	W14° 12' 28.199"	Skinner et al., Ref. 40
MD97-2121	2314	S40° 22' 935''	E177° 59' 68''	Skinner et al., Ref. 4
VM21-30	617	S1° 1' 59.999"	W89° 7' 0.001"	Stott et al., Ref. 41
KNR159-5-36GGC	1268	S27° 31' 0.001"	W46° 28' 0.001"	Tessin et al., Ref. 42
KNR159-5-17JPC	1627	S27° 42' 0"	W46° 28' 59.999"	Tessin et al., Ref. 42
KNR159-5-78GGC	1820	S27° 28' 59.999"	W46° 19' 59.999"	Tessin et al., Ref. 42
KNR159-5-33GGC	2082	S27° 34' 0.001"	W46° 10' 59.999"	Tessin et al., Ref. 42
KNR159-5-42JPC	2296	S27° 46' 0.001"	W46° 37' 59.999"	Tessin et al., Ref. 42
KNR159-5-30GGC	2500	S28° 7' 59.999"	W46° 4' 0.001"	Tessin et al., Ref. 42
CD159-10	1237	N62° 58' 31.199"	W17° 35' 22.2"	Thornalley et al., Ref. 43
CD159-12	1938	N62° 5' 25.8"	W17° 49' 10.801"	Thornalley et al., Ref. 43
CD159-15	2133	N62° 5' 25.8"	W17° 8' 2.4"	Thornalley et al., Ref. 43
CD159-17	2303	N61° 28' 54.001"	W19° 32' 9.6"	Thornalley et al., Ref. 43

Supplementary Table 2: General information of sediment cores used in this study.

Sediment Core	Water Depth (m)	Latitude	Longitude	Av. Sed. Rate (cm/kyr)	Region	Vessel Name	Remarks
PS75/104-1	835	S44° 46' 9.012"	E174° 31' 31.8"	7.5	NZM	Polarstern	
SO213-84-1	972	S 45° 7' 30.3"	E 174° 34' 55.32"	6	NZM	Sonne	
SO213-82-1	2066	S 45° 46' 39.18"	E 176° 36' 7.32"	2.5	NZM	Sonne	
PS75/100-4	2498	S 45° 45' 24.588"	E 177° 8' 55.788"	6	NZM	Polarstern	
SO213-79-2	3140	S 45° 50' 41.28"	E 179° 34' 9.72"	9.2	NZM	Sonne	
PS75/059-2	3613	S 54° 12' 54"	W 125° 25' 31.8"	3.5	EPR	Polarstern	
SO213-76-2	4339	S 46° 12' 59.88"	W 178° 1' 40.2"	22.5	NZM	Sonne	
MD97-2120	1210	S 45° 32' 3.599"	E 174° 55' 50.999"	16	NZM	Marion Dufresne	Reference core

Supplementary Table 3: Planktic and benthic radiocarbon dates for all cores. The benthic to planktic age reversals (B<P) can be eliminated if a reservoir correction is applied for the planktic data.

Sediment Core	Water Depth (m)	Modern Water Mass	Sample Depth (cm)	Species	14C Age	Error	Surf Res Age	D14C	Lab Code	Remarks
PS75/104-1	835	AAIW	18	G. bulloides	7763	110	782	-640.30	56144.2.1	
PS75/104-1	835	AAIW	18	Mixed benthics	7160	85		-612.70	56145.2.1	B<P
PS75/104-1	835	AAIW	58	G. bulloides	14073	134	1061	-843.30	56148.2.1	
PS75/104-1	835	AAIW	58	Mixed benthics	14567	132		-838.10	56149.1.1	
PS75/104-1	835	AAIW	68	G. bulloides	14592	134	357	-841.20	56150.2.1	
PS75/104-1	835	AAIW	68	Mixed benthics	15682	143		-859.60	56151.2.1	
PS75/104-1	835	AAIW	96	G. bulloides	15678	138	243	-860.60	56158.2.1	
PS75/104-1	835	AAIW	96	Mixed benthics	17066	166		-881.30	56159.1.1	
SO213-84-1	972	AAIW	55	G. bulloides	17700	70	856	-890.5	OS-106834	
SO213-84-1	972	AAIW	55	Mixed benthics	18600	210		-901.75	OS-107002	
SO213-84-1	972	AAIW	68	G. bulloides	20700	140	834	-924.5	OS-101445	
SO213-84-1	972	AAIW	68	Mixed benthics	20700	180		-924.41	OS-101628	B=P
SO213-84-1	972	AAIW	126	G. bulloides	25200	260	626	-957.04	OS-101446	
SO213-84-1	972	AAIW	126	Mixed benthics	25100	250		-956.13	OS-101447	B<P
SO213-82-1	2066	UCDW	10	G. bulloides	6680	55	533	-567.89	OS-102929	
SO213-82-1	2066	UCDW	10	Mixed benthics	6960	30		-582.7	OS-103138	
SO213-82-1	2066	UCDW	26	G. bulloides	8405	30	127	-648.8	UCIAMS-133187	
SO213-82-1	2066	UCDW	26	Mixed benthics	11040	40		-746.9	UCIAMS-133188	
SO213-82-1	2066	UCDW	38	G. bulloides	12350	55	490	-786.12	OS-102930	
SO213-82-1	2066	UCDW	38	Mixed benthics	13300	55		-810.35	OS-103140	
SO213-82-1	2066	UCDW	44	G. bulloides	12950	85	327	-802.48	OS-109596	
SO213-82-1	2066	UCDW	44	Mixed benthics	14550	85		-837.74	OS-109597	
SO213-82-1	2066	UCDW	54	G. bulloides	14850	65	340	-843.65	OS-103141	
SO213-82-1	2066	UCDW	54	Mixed benthics	16100	85		-865.95	OS-103142	
SO213-82-1	2066	UCDW	72	G. bulloides	18050	95	535	-895.18	OS-103143	
SO213-82-1	2066	UCDW	72	Mixed benthics	21400	230		-930.83	OS-104326	
SO213-82-1	2066	UCDW	90	G. bulloides	25600	110	893	-959.1	OS-106669	
SO213-82-1	2066	UCDW	90	Mixed benthics	26600	620		-963.97	OS-107006	
PS75/100-4	2498	UCDW	2	G. bulloides	3730	20	479	-371.6	UCIAMS-133181	
PS75/100-4	2498	UCDW	2	Mixed benthics	3740	20		-372.4	UCIAMS-133182	
PS75/100-4	2498	UCDW	12	G. bulloides	8670	30	848	-662.63	OS-98717	
PS75/100-4	2498	UCDW	12	Mixed benthics	8780	35		-667.33	OS-98709	
PS75/100-4	2498	UCDW	24	G. bulloides	10350	45	752	-725.86	OS-108460	
PS75/100-4	2498	UCDW	24	Mixed benthics	12050	50		-777.88	OS-108461	
PS75/100-4	2498	UCDW	26	G. bulloides	11900	45	520	-773.73	OS-98714	
PS75/100-4	2498	UCDW	26	Mixed benthics	12750	65		-796.73	OS-98710	
PS75/100-4	2498	UCDW	36	G. bulloides	13340	50	582	-810.0	UCIAMS-133183	
PS75/100-4	2498	UCDW	36	Mixed benthics	15850	60		-861.0	UCIAMS-133184	
PS75/100-4	2498	UCDW	54	G. bulloides	13500	50	428	-815.27	OS-98713	
PS75/100-4	2498	UCDW	54	Mixed benthics	19700	110		-914.4	OS-100171	
PS75/100-4	2498	UCDW	62	G. bulloides	14400	110	741	-834.74	OS-114549	
PS75/100-4	2498	UCDW	62	Mixed benthics	21800	90		-934.56	OS-112948	
PS75/100-4	2498	UCDW	68	G. bulloides	17650	150	857	-889.57	OS-108064	
PS75/100-4	2498	UCDW	68	Mixed benthics	25400	500		-957.88	OS-108071	
PS75/100-4	2498	UCDW	72	G. bulloides	18750	100	877	-903.1	UCIAMS-133185	
PS75/100-4	2498	UCDW	72	Mixed benthics	26700	260		-964.0	UCIAMS-133186	
PS75/100-4	2498	UCDW	74	G. bulloides	20400	170	903	-921.77	OS-101818	
PS75/100-4	2498	UCDW	74	Mixed benthics	26700	270		-964.2	OS-101819	
PS75/100-4	2498	UCDW	103	G. bulloides	27000	240	421	-965.73	OS-98715	
PS75/100-4	2498	UCDW	103	Mixed benthics	27500	250		-967.65	OS-98723	
SO213-79-2	3140	LCDW	26	G. bulloides	7070	30	926	-588.42	OS-100217	
SO213-79-2	3140	LCDW	26	Mixed benthics	8370	35		-650.06	OS-100669	
SO213-79-2	3140	LCDW	36	G. bulloides	8390	30	428	-650.81	OS-100566	
SO213-79-2	3140	LCDW	36	Mixed benthics	9650	40		-701.33	OS-100670	
SO213-79-2	3140	LCDW	54	G. bulloides	11900	40	367	-773.83	OS-100567	
SO213-79-2	3140	LCDW	54	Mixed benthics	13250	60		-809.38	OS-100671	
SO213-79-2	3140	LCDW	66	G. bulloides	13550	100	953	-816	OS-100866	
SO213-79-2	3140	LCDW	66	Mixed benthics	15100	90		-848	OS-100867	
SO213-79-2	3140	LCDW	104	G. bulloides	14000	110	455	-826.59	OS-104341	
SO213-79-2	3140	LCDW	104	Mixed benthics	18400	100		-899.65	OS-103135	
PS75/059-2	3613	LCDW	2	G. bulloides	3900	30	478	-389.3	OS-101322	
PS75/059-2	3613	LCDW	2	Mixed benthics	4030	45		-403.8	OS-101191	
PS75/059-2	3613	LCDW	17	G. bulloides	6680	45	463	-567.7	OS-101301	
PS75/059-2	3613	LCDW	17	Mixed benthics	7630	35		-615.9	OS-101192	
PS75/059-2	3613	LCDW	37	G. bulloides	10650	55	459	-736.2	OS-101248	
PS75/059-2	3613	LCDW	37	Mixed benthics	12000	55		-777.4	OS-101247	
PS75/059-2	3613	LCDW	47	G. bulloides	11950	45	678	-776.4	OS-101249	
PS75/059-2	3613	LCDW	47	Mixed benthics	15100	50		-848.2	OS-101250	
PS75/059-2	3613	LCDW	57	G. bulloides	14200	50	535	-830	OS-101252	
PS75/059-2	3613	LCDW	57	Mixed benthics	18400	65		-899.2	OS-101303	
PS75/059-2	3613	LCDW	59	G. bulloides	14800	60	453	-842.75	OS-105209	
PS75/059-2	3613	LCDW	59	Mixed benthics	19450	250		-911.96	OS-105426	
PS75/059-2	3613	LCDW	62	G. bulloides	20700	100	3456	-924.99	OS-107186	
PS75/059-2	3613	LCDW	62	Mixed benthics	25200	660		-956.89	OS-107702	
PS75/059-2	3613	LCDW	69	G. bulloides	28900	220	105	-972.77	OS-107187	
PS75/059-2	3613	LCDW	69	Mixed benthics	28900	700		-972.66	OS-107690	
PS75/059-2	3613	LCDW	77	G. bulloides	29500	130	374	-974.9	OS-101307	
PS75/059-2	3613	LCDW	77	Mixed benthics	30200	190		-977	OS-101308	

SO213-76-2	4339	LCDW + AABW	3	G. bulloides	2820	30	763	-301.22	OS-98841
SO213-76-2	4339	LCDW + AABW	3	Mixed benthics	3360	30		-346.87	OS-98842
SO213-76-2	4339	LCDW + AABW	43	G. bulloides	9410	35	1031	-692.55	OS-98843
SO213-76-2	4339	LCDW + AABW	43	Mixed benthics	10350	45		-726.75	OS-98847
SO213-76-2	4339	LCDW + AABW	67	G. bulloides	12270	40	711	-782.9	UCIAMS-133189
SO213-76-2	4339	LCDW + AABW	67	Mixed benthics	13240	50		-807.6	UCIAMS-133190
SO213-76-2	4339	LCDW + AABW	93	G. bulloides	13650	65	681	-818.62	OS-98844
SO213-76-2	4339	LCDW + AABW	93	Mixed benthics	15000	65		-846.66	OS-98845
SO213-76-2	4339	LCDW + AABW	149	G. bulloides	14700	75	490	-841.14	OS-105212
SO213-76-2	4339	LCDW + AABW	149	Mixed benthics	16450	75		-872.3	OS-105213
SO213-76-2	4339	LCDW + AABW	203	G. bulloides	15000	190	397	-846.78	OS-104351
SO213-76-2	4339	LCDW + AABW	203	Mixed benthics	17000	95		-880.74	OS-103137
SO213-76-2	4339	LCDW + AABW	249	G. bulloides	16000	120	306	-864.73	OS-105410
SO213-76-2	4339	LCDW + AABW	249	Mixed benthics	17400	80		-886.42	OS-105214
SO213-76-2	4339	LCDW + AABW	313	G. bulloides	17850	250	719	-892.33	OS-98988
SO213-76-2	4339	LCDW + AABW	313	Mixed benthics	20900	120		-926.58	OS-98846
SO213-76-2	4339	LCDW + AABW	491	G. bulloides	19900	200	567	-916.65	OS-105408
SO213-76-2	4339	LCDW + AABW	491	Mixed benthics	22700	190		-941.03	OS-104471
SO213-76-2	4339	LCDW + AABW	515	G. bulloides	21900	190	823	-935.2	OS-102409
SO213-76-2	4339	LCDW + AABW	515	Mixed benthics	24200	1100		-951.38	OS-103299
SO213-76-2	4339	LCDW + AABW	662	G. bulloides	23500	460	831	-947.06	OS-104708
SO213-76-2	4339	LCDW + AABW	662	Mixed benthics	25700	270		-959.33	OS-104472

Supplementary Table 4: Mean major element composition of glass shards from cores SO213-79-2 and SO213-76-2 and selected onshore and offshore Kawakawa/Oruanui Tephra (KOT) correlatives.

	SiO_2	Al_2O_3	TiO_2	FeO	MgO	MnO	CaO	Na_2O	K_2O	Cl	H_2O	*n*	Reference
**SO213-79-2**<br>(150-151 cm)	78.33 (0.25)	12.51 (0.10)	0.12 (0.01)	1.20 (0.08)	0.12 (0.02)	0.05 (0.04)	1.06 (0.06)	3.65 (0.17)	2.95 (0.10)	0.02 (0.01)	4.81 (0.94)	19	
**SO213-76-2**<br>(507-508 cm)	78.31 (0.22)	12.50 (0.12)	0.12 (0.01)	1.24 (0.08)	0.11 (0.01)	0.04 (0.03)	1.04 (0.09)	3.59 (0.21)	3.03 (0.42)	0.02 (0.01)	4.79 (1.00)	19	
**TAN0513-14**<br>(43-44 cm)	78.50 (0.26)	12.55 (0.11)	0.12 (0.02)	1.19 (0.06)	0.12 (0.01)	0.05 (0.02)	1.00 (0.06)	3.42 (0.15)	3.07 (0.16)	nd [b]	3.87 (1.09)	46	Ryan et al., Ref. 14
**Okarito Lagoon**<br>south Westland	78.04 (0.30)	12.65 (0.15)	0.14 (0.03)	1.13 (0.10)	0.13 (0.02)	0.06 (0.06)	1.02 (0.07)	3.34 (0.41)	3.27 (0.34)	0.20 (0.05)	5.05 (1.42)	20	Newnham et al., Ref. 15
**Galway Tarn**<br>south Westland	77.75 (0.36)	12.59 (0.16)	0.13 (0.03)	1.13 (0.13)	0.13 (0.03)	0.08 (0.05)	1.04 (0.06)	3.79 (0.24)	3.06 (0.11)	0.23 (0.16)	4.40 (1.62)	20	Newnham et al., Ref. 15
**AT-331, ODP Site 1123**<br>1.01 mcd	77.98 (0.29)	12.31 (0.12)	0.13 (0.03)	1.16 (0.08)	0.11 (0.02)	nd	1.07 (0.11)	3.98 (0.10)	3.05 (0.19)	0.15 (0.02)	4.23 (1.02)	15	Alloway et al., Ref. 16
**Whangamata Road, TS**<br>50334-40 (T17/619830)	79.01 (0.38)	12.15 (0.30)	0.13 (0.03)	1.15 (0.19)	0.12 (0.02)	nd	1.05 (0.19)	3.31 (0.21)	3.07 (0.25)	nd	5.65 (1.62)	74	Villamor et al., 2007 Ref. 17

Supplementary References

1 Schlitzer, R. Ocean Data View v. 4.7.2. (owa.awi.de, 2015).

2 Sikes, E. L., Samson, C. R., Gullderson, T. P. & Howard, W. R. Old radiocarbon ages in the southwest Pacifc Ocean during the last glacial period and deglaciation. *Nature* **405**, 555-559 (2000).

3 Rose, K. A. *et al.* Upper-ocean-to-atmosphere radiocarbon offsets imply fast deglacial carbon dioxide release. *Nature* **466**, 1093-1097 (2010).

4 Skinner, L. C. *et al.* Reduced ventilation and enhanced magnitude of the deep Pacific carbon pool during the last glacial period. *Earth and Planetary Science Letters* **411**, 45-52 (2015).

5 Orsi, A. H., Whitworth III, T. & Nowlin Jr, W. D. On the meridional extent and fronts of the Antarctic Circumpolar Current. *Deep-Sea Research I* **42**, 641-673 (1995).

6 Cook, M. S. & Keigwin, L. D. Radiocarbon profiles of the NW Pacific from the LGM and deglaciation: evaluating ventilation metrics and the effect of uncertain surface reservoir ages. *Paleoceanography* (2015).

7 Adkins, J. F. & Boyle, E. A. Changing atmospheric $\Delta^{14}C$ and the record of deepwater paleoventilation ages. *Paleoceanography* **12**, 337-344 (1997).

8 Bintanja, R., van de Wal, R. S. W. & Oerlemans, J. Modelled atmospheric temperatures and global sea levels over the past million years. *Nature* **437**, 125-128 (2005).

9 Key, R. M. *et al.* A global ocean carbon climatology: Results from Global Data Analysis Project (GLODAP). *Global Biochemical Chycles* **18**, 1-23 (2004).

10 Pahnke, K., Zahn, R., Elderfield, H. & Schulz, M. 340,000-Year Centennial-Scale Marine Record of Southern Hemisphere Climatic Oscillation. *Science* **301**, 948-952 (2003).

11 EPICA Community Members. One-to-one coupling of glacial climate variability in Greenland and Antarctica. *Nature* **444**, 195-198 (2006).

12 Veres, D. *et al.* The Antarctic ice core chronology (AICC2012): an optimized thousand yearsmulti-parameter and multi-site dating approach for the last 120. *Climate of the Past* **9**, 1773-1748 (2013).

13 Pahnke, K. & Zahn, R. Southern Hemisphere Water Mass Conversion Linked with North Atlantic Climate Variability. *Science* **307**, 1741-1746 (2005).

14 Ryan, M. T. *et al.* Vegetation and climate in Southern Hemisphere mid-latitudes since 210 ka: new insights from marine and terrestrial pollen records from New Zealand. *Quaternary Science Reviews* **48**, 80-98 (2012).

15 Newnham, R. M., Vandergoes, M. J., Hendy, C. H., Lowe, D. J. & Preusser, F. A terrestrial palynological record for the last two glacial cycles from southwestern New Zealand. *Quaternary Science Reviews* **26**, 517-535 (2007).

16 Alloway, B. V., Pillans, B., Carter, L., Naish, T. R. & Westgate, J. A. Onshore–offshore correlation of Pleistocene rhyolitic eruptions from New Zealand: implications for TVZ eruptive history and paleoenvironmental construction. *Quaternary Science Reviews* **24**, 1601-1622 (2005).

17 Villamor, P., Van Dissen, R., Alloway, B. V., Palmer, A. S. & Lichfield, N. The Rangipo fault, Taupo rift, New Zealand: An example of temporal slip-rate and single-event displacement variability in a volcanic environment. *Geological Society of America Bulletin* **119**, 529-547 (2007).

18 Broecker, W. & Clark, E. Search for a glacial-age ^{14}C-depleted ocean reservoir. *Geophysical Research Letters* **37**, 1-6 (2010).

19 Barker, S., Knorr, G., Vautravers, M. J., Diz, P. & Skinner, L. C. Extreme deepening of the Atlantic overturning circulation during deglaciation. *Nature Geoscience* **3**, 567-571 (2010).

20 Basak, C., Martin, E. E., Horikawa, K. & Marchitto, T. M. Southern Ocean source of ^{14}C-depleted carbon in the North Pacific Ocean during the last deglaciation. *Nature Geoscience* **3**, 770-773 (2010).

21 Bostock, H. C., Opdyke, B. N., Gagan, M. K. & Fifield, L. K. Carbon isotope evidence for changes in Antarctic Intermediate Water circulation and ocean ventilation in the southwest Pacific during the last deglaciation. *Paleoceanography* **19**, 1-15 (2004).

22 Böning, C. W., Dispert, A., Visbeck, M., Rintoul, S. R. & Schwarzkopf, F. U. The response of the Antarctic Circumpolar Current to recent climate change. *Nature Geoscience* **1**, 864-869 (2008).

23 Bryan, S. P., Marchitto, T. M. & Lehman, S. J. The release of ^{14}C-depleted carbon from the deep ocean during the last deglaciation: Evidence from the Arabian Sea. *Earth and Planetary Science Letters* **298**, 244-254 (2010).

24 Burke, A. & Robinson, L. F. The Southern Ocean's Role in Carbon Exchange During the Last Deglaciation. *Science* **335**, 557-561 (2012).

25 Cléroux, C., deMenocal, P. & Guilderson, T. P. Deglacial radiocarbon history of tropical Atlantic thermocline waters: absence of CO_2 reservoir purging signal. *Quaternary Science Reviews* **30**, 1875-1882 (2011).

26 De Pol-Holz, R., Keigwin, L. D., Southon, J., Hebbeln, D. & Mohtadi, M. No signature of abyssal carbon in intermediate waters off Chile during deglaciation. *Nature Geoscience* **3**, 192-195 (2010).

27 De Pol-Holz, R., Mohtadi, M. & Southon, J. R. in *EGU General Assembly* (Vienna, 2012).

28 Galbraith, E. D. *et al.* Carbon dioxide release from the North Pacific abyss during the last deglaciation. *Nature* **449**, 890-894 (2007).
29 Goldstein, S. J., Lea, D. W., Chakraborty, S., Kashgarian, M. & Murrell, M. T. Uranium-series and radiocarbon geochronology of deep-sea corals: implications for Southern Ocean ventilation rates and the oceanic carbon cycle. *Earth and Planetary Science Letters* **193**, 167-182 (2001).
30 Keigwin, L. D. Radiocarbon and stable isotope constraints on Last Glacial Maximum and Younger Dryas ventilation in the western North Atlantic. *Paleoceanography* **19**, 1-15 (2004).
31 Robinson, L. F. *et al.* Radiocarbon Variability in the Western North Atlantic During the Last Deglaciation. *Science* **310**, 1469-1473 (2005).
32 Lund, D. C., Mix, A. C. & Southon, J. Increased ventilation age of the deep northeast Pacific Ocean during the last deglaciation. *Nature Geoscience* **4**, 771-774 (2011).
33 Mangini, A. *et al.* Deep sea corals off Brazil verify a poorly ventilated Southern Pacific Ocean during H2, H1 and the Younger Dryas. *Earth and Planetary Science Letters* **293**, 269-276 (2010).
34 Marchitto, T. M., Lehman, S. J., Ortiz, J. D., Flückinger, J. & van Geen, A. Marine Radiocarbon Evidence for the Mechanism of Deglacial Atmospheric CO_2 Rise. *Science* **316**, 1456-1459 (2007).
35 Rae, J. W. B. *et al.* Deep water formation in the North Pacific and deglacial CO_2 rise. (2014).
36 Robinson, L. F. & van de Flierdt, T. Southern Ocean evidence for reduced export of North Atlantic Deep Water during Heinrich event 1. *Geology* **37**, 195-198 (2009).
37 Romahn, S., Mackensen, A., Groeneveld, J. & Pätzold, J. Deglacial intermediate water reorganization: new evidence from the Indian Ocean. *Climate of the Past* **10**, 293-303 (2014).
38 Sarnthein, M., Grootes, P. M., Kennet, J. P. & Nadeau, M.-J. in *Ocean Circulation: Mechanisms and Impacts—Past and Future Changes of Meridional Overturning* Vol. 173 (eds A. Schmittner, J. C. H. Chiang, & S. R. Hemming) 175-196 (AGU, 2007).
39 Skinner, L. C. & Shackleton, N. J. Rapid transient changes in northeast Atlantic deep water ventilation age across Termination I. *Paleoceanography* **19**, 1-11 (2004).
40 Skinner, L. C., Fallon, S., Waelbroeck, C., Michel, E. & Barker, S. Ventilation of the Deep Southern Ocean and Deglacial CO_2 Rise. *Science* **328**, 1147-1151 (2010).
41 Stott, L. D., Southon, J., Timmermann, A. & Koutavas, A. Radiocarbon age anomaly at intermediate water depth in the Pacific Ocean during the last deglaciation. *Paleoceanography* **24**, 1-10 (2009).
42 Tessin, A. C. & Lund, D. C. Isotopically depleted carbon in the mid-depth South Atlantic during the last deglaciation. *Paleoceanography* **28**, 296-306 (2013).
43 Thornalley, D. J. R., Elderfield, H. & McCave, I. N. Intermediate and deep water paleoceanography of the northern North Atlantic over the past 21,000 years. *Paleoceanography* **25**, 1-17 (2010).
